# Combined Metallomics/Transcriptomics Profiling Reveals a Major Role for Metals in Wound Repair

**DOI:** 10.3389/fcell.2021.788596

**Published:** 2021-11-30

**Authors:** Holly N. Wilkinson, Barbara-Ann Guinn, Matthew J. Hardman

**Affiliations:** ^1^ Centre for Atherothrombosis and Metabolic Disease, Hull York Medical School, The University of Hull, Hull, United Kingdom; ^2^ Department of Biomedical Sciences, Faculty of Health, The University of Hull, Hull, United Kingdom

**Keywords:** metallome, wound healing, skin, RNA-sequencing, calcium, metals

## Abstract

Endogenous metals are required for all life, orchestrating the action of diverse cellular processes that are crucial for tissue function. The dynamic wound healing response is underpinned by a plethora of such cellular behaviours, occurring in a time-dependent manner. However, the importance of endogenous metals for cutaneous repair remains largely unexplored. Here we combine ICP-MS with tissue-level RNA-sequencing to reveal profound changes in a number of metals, and corresponding metal-regulated genes, across temporal healing in mice. Wound calcium, magnesium, iron, copper and manganese are elevated at 7 days post-wounding, while magnesium, iron, aluminium, manganese and cobalt increase at 14 days post-wounding. At the level of transcription, wound-induced pathways are independently highly enriched for metal-regulated genes, and vice versa. Moreover, specific metals are linked to distinct wound-induced biological processes and converge on key transcriptional regulators in mice and humans. Finally, we reveal a potential role for one newly identified transcriptional regulator, TNF, in calcium-induced epidermal differentiation. Together, these data highlight potential new and diverse roles for metals in cutaneous wound repair, paving the way for further studies to elucidate the contribution of metals to cellular processes in the repair of skin and other tissues.

## Introduction

Skin is the first barrier to noxious environmental onslaught and as such, must be repaired quickly and efficiently when damaged. This wound healing process involves a cascade of cellular signalling events that actuate functional responses to confer tissue repair (Wilkinson and Hardman, 2020). These events are grouped into characteristic wound healing stages (haemostasis, inflammation, proliferation and extracellular matrix remodelling) that are tightly controlled but remain highly dynamic and often overlap (Greaves et al., 2013). Despite a high level of regulation and redundancy, aberrations to normal skin repair are surprisingly frequent. Over-exuberant healing leads to excessive scarring and keloids, while insufficient healing results in infection and non-healing “chronic” wounds. Chronic wounds remain a major socio-economic burden, with existing treatments often inadequate (Frykberg and Banks, 2015). Thus, there is an ongoing need to fully understand the molecular and cellular aspects of normal healing, in order to effectively address healing pathology.

Metallomics, the global characterisation of metals and metalloids in biological systems (Haraguchi, 2017), is a rapidly emerging field. When combined with existing proteomics, genomics or metabolomics approaches, it can provide an integrated, systematic understanding of biology (Singh and Verma, 2018). Metals are fundamental for almost all biological processes including metabolism, energy transduction, gene expression and cell signalling (Yannone et al., 2012). This is best demonstrated by the fact that almost half of all enzymes contain a transition metal core (e.g., copper, zinc or iron) that is necessary for appropriate enzymatic function (Andreini et al., 2008). Paradoxically, despite their clear biological importance, current understanding of the temporal and spatial distribution of metals in tissues, and subsequent integration with other omics data, remains fairly limited.

There is existing literature pertaining to the importance of specific endogenous metals in skin biology and wound healing. Calcium flashes occur within seconds to trigger a healing response (Razzell et al., 2013), while calcium is also widely reported to regulate epidermal stratification and barrier integrity (Elias et al., 2002), platelet activation (Elaïb et al., 2016), cellular migration (Mandeville and Maxfield, 1997; Wei et al., 2009), macrophage phagocytosis (Vashi et al., 2017) and cellular proliferation (Berridge, 1995; Schwarz et al., 2006). Each of these processes is crucial for effective skin wound healing. Wound-relevant roles for other metals are also emerging, such as iron, which is involved in haemostasis (Pretorius et al., 2013), inflammation (Haldar et al., 2014), and extracellular matrix (ECM) deposition and remodelling (Wilkinson et al., 2019b; 2019c). Given that these reductionist studies have revealed important mechanistic roles for individual metals in specific repair processes, it is somewhat surprising that little attempt has been made to systematically characterise the temporal distribution of metals across the wound repair continuum, and globally integrate these data with molecular and cellular level changes.

In the present study, we show for the first time that temporal alterations in the wound metallome correlate with both metal- and injury-linked transcriptional changes across normal murine skin repair. Specifically, wound-induced genes are highly enriched for metal functional annotation, clustering into particular biological processes that correlate with hitherto unreported temporal changes in wound metal distribution. Our findings are corroborated further by exploration of metal-linked transcriptional changes in human wounds.

## Materials and Methods

### *In Vivo* Wound Healing

Female C57BL/6J mice (8–10 weeks old) were purchased (Envigo Ltd., United Kingdom) and housed at the Biological Services Facility (The University of Manchester, United Kingdom). Animal experimentation was performed under full United Kingdom Home Office approval (project licence: 70/8136). Food and water was given *ad libitum* and cages were kept under a constant 12-h light-dark cycle. Mice were anaesthetised and 2 mm × 6 mm full thickness excisional dorsal wounds were created using trace metal free instruments. Wounds were positioned 2 cm caudal to the base of the skull, 0.5 cm either side of the midline. Mice were administered buprenorphine post operatively and wounds were left to heal via secondary intention. Skin (D0) and wounds were collected at days one (D1), three (D3), seven (D7) and fourteen (D14) post-wounding (*n* = 5 mice per time point). Wound samples were carefully dissected to include the full wound bed and 3–4 mm of peri-wound tissue. Skin and wounds were flash frozen in liquid nitrogen and stored at −80°C prior to ICP-MS and RNA isolation or fixed in neutral buffered formalin for histological assessment.

### Metal Analysis

Frozen tissue was freeze dried and digested in a 50:50 mixture of 30% hydrogen peroxide (Sigma-Aldrich, Dorset, United Kingdom) and trace metal free nitric acid (Thermo Fisher Scientific, Loughborough, United Kingdom) in a MARS 6 microwave with a MARSXPRESS™ vessel (CEM Microwave Technology Ltd, Buckingham, United Kingdom) as previously described (Wilkinson et al., 2019c). DOLT-5 reference material (National Research Council, Canada) was prepared and analysed alongside digested tissue samples. Internal standards (Rh at ∼100 ppb) were used as calibration and a water reference standard (CRM 1643E) was included for internal standard verification. Samples were run on an Agilent 7800 inductively coupled plasma mass spectrometer (Agilent Technologies, Cheshire, United Kingdom). The operating conditions were: Carrier gas flow of 1.05 L/min and spray chamber temperature of 2°C; plasma mode was general purpose, RF forward power was 1,550 W with a sampling depth of 10 mm; He cell gas flow of 5 ml/min and Ni cones; the extraction lens was 1 V, with kinetic energy discrimination of 5 V. Major isotopes of the elements were monitored, with a mass to charge = 1. Working conditions were checked at the start of the day by injecting the standard solution of several isotopes at 1 ppb level, tuned and the ionisation signal compared to the previous day. The relative standard deviation among replicates was evaluated as standard. A precision of <5% with sensitivity of ∼ppb was routinely achieved.

### Histology

Sections (5 µM thick) were taken from paraffin embedded blocks. Slides were dewaxed and brought to dH_2_O down an ethanol gradient. Masson’s trichrome staining, and immunoperoxidase staining for neutrophils and macrophages, was performed as described in Wilkinson et al. (2019a). For immunofluorescent staining, antigen retrieval was achieved with citrate buffer and sections blocked in goat serum and M.O.M block (Vector Laboratories, CA, United States). Rabbit anti-keratin 14 (clone: Poly19053; Biolegend, CA, United States), mouse anti-TNF-α (clone: 52B83; Abcam, Cambridge, United Kingdom) and rat anti-CD107b (clone: M3/84; BD Biosciences, NJ, United States) primary antibodies were detected with Alexa Fluor conjugated secondary antibodies (Thermo Fisher Scientific) and slides were mounted with MOWIOL 488 containing DAPI (Thermo Fisher Scientific). Slides were imaged on an LSM 710 confocal laser scanning microscope (Carl Zeiss, Oberkochen, Germany) using a 20× objective lens and 405-nm diode, 488-nm argon, and 561-nm diode-pumped solid-state lasers. Staining was quantified using ImageJ v.1.8.0 (National Institutes of Health, MD, United States).

### Extraction of UniProt Metal Lists

To initially investigate the link between wound transcriptional changes and metals, we extracted lists of metal-linked genes from the UniProt Knowledge Base—a collated resource providing comprehensive protein functional annotation. UniProt entries are tagged with keywords to enable retrieval of specific protein subsets. To extract metal-associated entries, we retrieved subsets using the following keywords: calcium (KW-0106); magnesium (KW-0460); iron (KW-0408); zinc (KW-0862); copper (KW-0186) and; manganese (KW-0464). To be included in a metal keyword list, proteins must bind at least one metal atom, or be functionally dependent on the metal in question. Each metal keyword list was next filtered to include only reviewed annotations, and then filtered by species of interest (e.g., mouse for RNA-Seq data and human for microarray comparison). Finally, gene name entries for each protein were extracted for functional annotation and overrepresentation analysis (described below).

### RNA Isolation

RNA was extracted from skin, wounds and keratinocytes. Tissue was homogenised (T10 ULTRA-TURRAX^®^, IKA, Oxford, United Kingdom), and cells vortexed, in TRIzol^®^ reagent (Thermo Fisher Scientific) prior to chloroform phase separation. RNA was purified and eluted using the PureLink™ RNA Mini Kit (Thermo Fisher Scientific) following manufacturer’s instructions.

### RNA Sequencing

RNA-Sequencing (RNA-Seq) was achieved using the paired-end method and Illumina platform. Error rate distribution among reads was <0.04%, while over 90% of reads from each sample passed quality control. The sequenced library was aligned to the reference genome using HISAT2 and assembled to map the transcriptome (service provided by Novogene Ltd., Cambridge, United Kingdom).

Differential expression analysis was performed on RNA-Seq count data using “DESeq2” (Love et al., 2014) in R.v.3.6.1 (R Core Team, 2020) with LFC shrinkage (Zhu et al., 2019). Genes were deemed differentially expressed when expression between experimental groups was above 1.5-fold different (log_2_ = 0.58), with 5% alpha level significance following multiple correction to reduce false discovery rate (Benjamini-Hochberg procedure). PC analysis was performed following variance stabilising transformation of count data. For hierarchal clustering, the top 250 most significant genes were filtered from transformed counts and clustered using Euclidian distance and Ward D2’s method within R package “gplots” (Warnes et al., 2020).

Overlap in gene subsets was determined in InteractiVenn (Heberle et al., 2015), while volcano plots were made from gene lists using the R package “EnhancedVolcano” (Blighe, 2019). For functional annotation, Ensembl gene identifiers were scrutinised in the Database for Annotation, Visualisation and Integrated Discovery v.6.8 (Huang et al., 2009), with top relevant annotation (gene ontology, KEGG pathway, UniProt KB) plotted against Benjamini-Hochberg adjusted *p* value. Markov clustering was performed on count data filtered for the combined list of upregulated DEGs using the Graphia tool (Kajeka Ltd., United Kingdom). Here, Pearson’s correlation for similarity was set to 0.94 and nodes clustered using the Markov clustering algorithm based on expression profiles. Gene clusters were manually annotated using functional annotation (as in Nirmal et al., 2018). Metal overrepresentation was determined using software to calculate the significance in overlap between two sets of genes (see Plaisier et al., 2010). Ingenuity Pathway Analysis (Qiagen, Manchester, United Kingdom) was used to determine top upstream regulators in specifically identified clusters.

### Quantitative Real-Time qPCR (qRT-PCR)

Bioscript™ (Bioline, London, United Kingdom) and random primers (Promega, Southampton, United Kingdom) were used for reverse transcription of purified RNA. qRT-PCR was performed on a CFX Connect™ thermocycler (Bio-Rad, Hertfordshire, United Kingdom) with Takyon™ SYBR mastermix (Eurogentec, Hampshire, United Kingdom). Primer sequences for RNA-Seq validation and keratinocyte experiments are provided in Supplementary Table S1.

### Keratinocyte Experiments

Keratinocytes were isolated from mouse and human skin for TNFα inhibitor experiments. Human skin was collected from Castle Hill Hospital (Cottingham, Hull, United Kingdom) under full, informed patient consent and institutional approval (LREC: 17/SC/0220). Human and mouse skin was incubated in 0.2% Dispase II (Merck Life Science United Kingdom Ltd., Dorset, United Kingdom) at 4C overnight to separate epidermis and dermis. The epidermis was then dissociated in 0.25% Trypsin (Thermo Fisher Scientific). Trypsin was neutralized with heat-inactivated Foetal Bovine Serum (Thermo Fisher Scientific) and the cell suspensions pelleted. Human epidermal keratinocytes (HEKs) were cultured in Epilife media containing Epilife defined growth supplement on coating matrix (Thermo Fisher Scientific). Mouse epidermal keratinocytes (MEKs) were cultured in CnT-07 medium (CELLnTEC, Bern, Switzerland) on collagen IV (Corning, NY, United States). Confluent keratinocytes were scratched with a 1 ml filter tip to induce a wounding response. Treatment conditions were low calcium (Epilife or CNT07 alone), high calcium (media plus 2mM CaCl_2_) and high calcium with TNFα inhibitor (10 μM C87, Biotechne, Oxford, United Kingdom). HEKs (*n* = 3 donors across three independent experiments) and MEKs (*n* = 5 pooled, three independent experiments) were collected for qRT-PCR.

### Statistical Analysis

Mean ± standard deviations of the mean (SEM) were used for non-transcriptional data. One-way ANOVAs were performed on this data with Tukey’s *post-hoc* using R. v.3.6.1. Significance was determined at the alpha 0.05 level. Pearson’s correlation was performed on RNA-Seq qRT-PCR validation data using R. v.3.6.1.

## Results

### Global Profiling Reveals Temporal Changes in the Wound Metallome During Normal Repair

**FIGURE 1:**
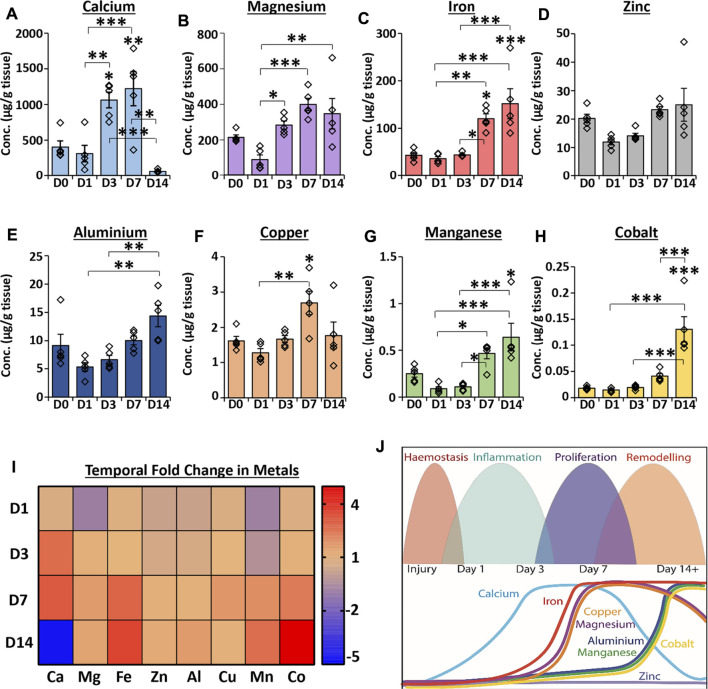
Temporal profiling reveals global changes in the metallome throughout normal wound repair. ICP-MS was used to measure metal abundance in skin and acute healing wounds. Absolute abundance of individual metals in tissue **(A–H)**. Heat map relative to normal skin (D0) where the saturated red value is 7 **(I)**. D1 = day 1 post-wounding. Schematic showing temporal metal changes alongside the main wound healing phases **(J)**. Mean ± SEM. *n* = 5 mice per group. One-way ANOVA with Tukey’s *post-hoc* analyses were performed. * = *p* < 0.05, ** = *p* < 0.01 and *** = *p* < 0.001. Asterisk alone versus D0.

Inductively coupled plasma mass spectrometry (ICP-MS), a highly sensitive elemental analysis technique (Lee et al., 2014), was used to measure metal abundance across an acute wound healing time-course. Tissue concentrations of eight skin-relevant metal elements were compared between unwounded skin (D0) and wounds at days 1, 3, 7 and 14 (D1, D3, D7 and D14) post-wounding (PW; Figure 1; absolute values provided in Supplementary Table S2). Healing stages were confirmed *via* histological analysis (Supplementary Figure S1).

Calcium levels were significantly elevated at D3 (*p* < 0.05) and D7 (*p* < 0.01) PW compared to D0 but dropped substantially at D14 PW to levels lower than observed at D0 (*p* < 0.01 versus D7; Figure 1A). Although magnesium concentration was not significantly altered between wounds and skin (Figure 1B), levels increased at D3 (versus D1; *p* < 0.05) and remained higher than D1 wounds throughout the 14 day period. Iron was substantially elevated at D7 and D14 versus D0 (Figure 1C, as previously reported in Wilkinson et al., 2019c), while zinc levels were not altered throughout the wound healing time course (Figure 1D). Aluminium (Figure 1E) was not significantly changed between skin and wounds, however levels at D14 were increased versus D1 (*p* < 0.01) and D3 (*p* < 0.01). Copper (Figure 1F) was highest at D7 PW (compared to D0, *p* < 0.05, and D1, *p* < 0.01). By contrast, manganese (*p* < 0.05; Figure 1G) and cobalt (*p* < 0.001; Figure 1H) showed the greatest elevation in levels at D14. The relative fold change in each metal versus D0 is shown in a heat map (Figure 1I), while the temporal profile for each metal is illustrated against specific stages of wound repair in Figure 1J. Collectively, these data demonstrate clear temporal changes in endogenous metals throughout normal murine wound healing.

### Independent of Context, Metal-Linked Genes Are Highly Annotated for Wound-Associated Biological Processes

**FIGURE 2:**
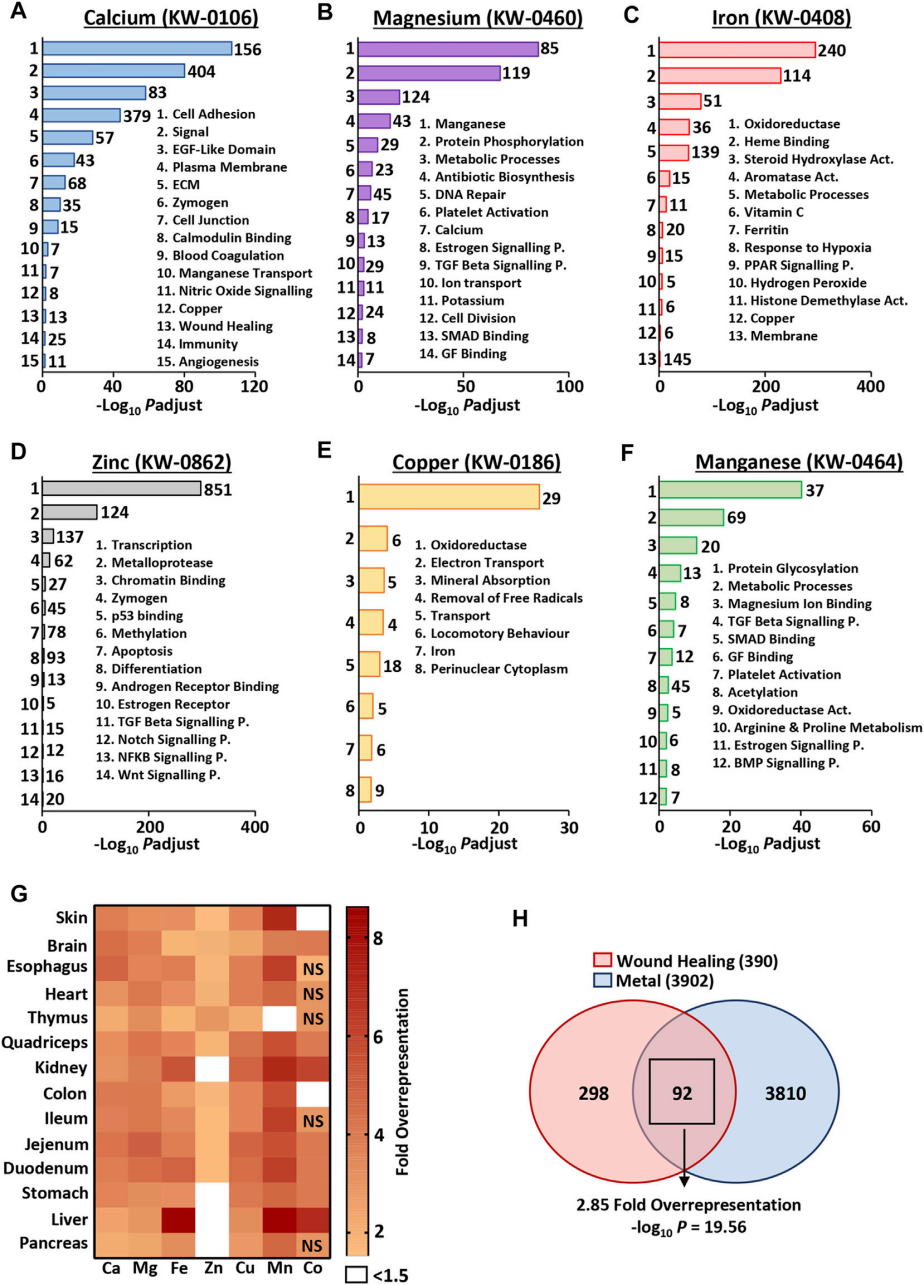
Metal-specific genes are associated with biological processes independent of context. Functional annotation was performed on metal gene lists from the UniProt knowledge base [KB **(A–F)**], demonstrating high annotation of processes linked to skin and wound healing. KW, keyword identifier; Numbers of genes, left of bars; ECM, extracellular matrix; P., pathway; Act., activity. The top 1,000 expressed skin and organ genes show differential overrepresentation of UniProt KB metal genes [observed vs expected fold change **(G)**]. Metal genes (combined UniProt KB list) are significantly overrepresented in the wound healing gene ontology group [GO: 0042060; **(H)**]. NS, non-significant; *P*adjust, Benjamini-Hochberg adjusted *p* value.

As metals were temporally altered throughout normal wound repair, we next asked whether metal-linked genes were independently linked to skin- and wound-associated pathways and processes (i.e., would there be global association between the metallome and transcriptome). Specifically, “metal” gene lists were extracted from the UniProt Knowledge Base (KB) and scrutinised by functional annotation analysis to identify overrepresented skin- and wound-related biological processes (Figures 2A–F). Here, genes from the (mouse-specific) calcium (KW-0106), magnesium (KW-0460), iron (KW-0408), zinc (KW-0862), copper (KW-0186) and manganese (KW-0464) UniProt KB lists were highly annotated for many wound-linked functional annotation terms, in a metal-specific manner (Supplementary Table S3). For example, cell adhesion (UniProt KB: KW-0130) and ECM (UniProt KB: KW-0272) annotations were overrepresented only in the calcium (KW-0106) gene list, while platelet activation (KEGG pathway: mmu04611) was highly enriched in magnesium (KW-0460) and manganese (KW-0464) lists.

Given the strong context independent correlation between metal-linked genes and wound-associated processes, we then asked whether metal-linked genes were overrepresented in specific murine tissues; skin (our data) and other organs (Söllner et al., 2017). To do this, we evaluated the number of metal-linked genes found in the top 1,000 expressed genes (based on RNA-Seq count) for each organ. In most cases, we observed more UniProt KB metal genes than would be expected by chance, with clear organ-specific differences in relative metal overrepresentation (Figure 2G; Supplementary Table S4). For example, in the skin, genes restricted to the manganese UniProt KB list were >7 fold overrepresented, while genes in the cobalt UniProt KB list displayed <1.5 fold overrepresentation. In line with known biological association (Graham et al., 2007), the most overrepresented (>8 fold) metal UniProt KB list across all organs was iron in the liver.

At an even broader level, genes linked to any one of the seven UniProt metals (combined UniProt KB lists: KW-0106, KW-0460, KW-0408, KW-0862, KW-0186, KW-0464 and KW-0170) showed significant overlap with known wound healing genes (GO group: 0042060; Figure 2H). Here, 92 out of 390 wound healing genes (23.6%) were also metal-linked, a 2.85-fold overrepresentation (−log_10_ *p* = 19.56; Supplementary Table S5). Taken together, these data clearly show a hitherto unappreciated convergence of genes associated with both metals and wounds. Indeed, metal-linked genes are overrepresented in the most highly expressed genes across a range of biological tissues, including the skin.

### RNA-Seq Profiling Reveals Temporal Alterations in Tissue Transcriptional Profiles Throughout Wound Repair

**FIGURE 3:**
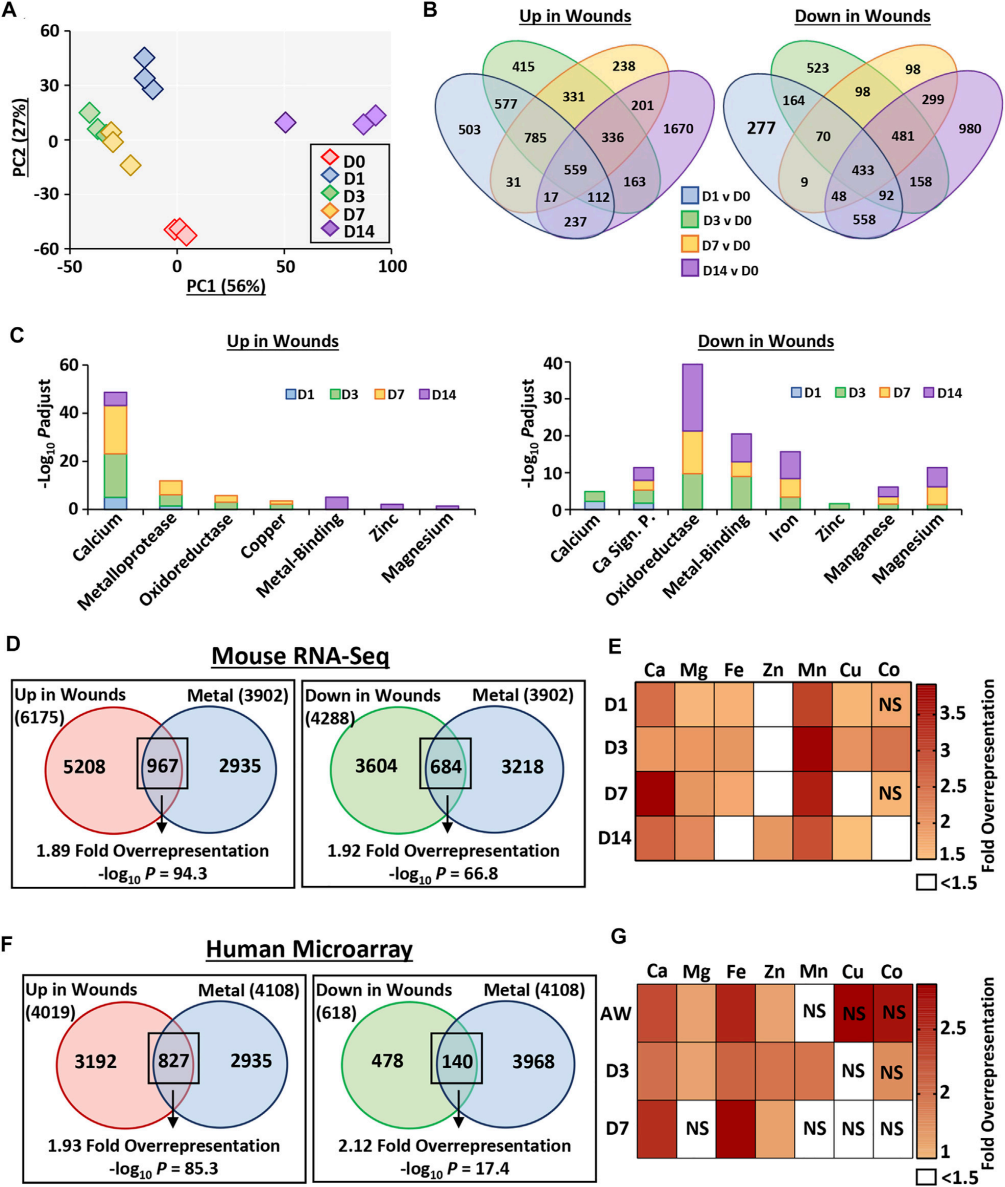
Metal-linked genes are considerably overrepresented in mouse and human wound transcriptomes. Murine wounds were collected at days 1, 3, 7 and 14 post-wounding for RNA-Sequencing. D0 = normal skin. *n* = 3 mice per group. Principal component (PC) analysis **(A)**. Percentage (%) contribution. Venn diagrams show differentially expressed genes (DEGs) in wounds versus D0. Log_2_ fold change ≥0.58 and Benjamini-Hochberg adjusted *p* value (*P*adjust) < 0.05 **(B)**. DEGs linked to metals by functional annotation **(C)**. Overrepresentation analysis demonstrates metal-linked (UniProt KB) genes are also injury-associated DEGs **(D)**. Wound-upregulated DEGs further separated by metal and time-point in **(E)**. Metal enrichment was confirmed in DEGs upregulated in human wounds *via* microarray **(F–G)**. AW, acute wound; D, day post-injury; NS, non-significant.

We performed detailed RNA-Seq profiling of gene expression change across normal murine healing, followed by in-depth analysis to explore the contribution of metals across the wound transcriptome (flow chart of approach provided in Supplementary Figure S2). RNA-Seq identified 10,294 differentially expressed genes (DEGs) between wounds and skin. Principal component analysis demonstrated clear clustering of individual samples by healing time-point, with some overlap between D3 and D7 (Figure 3A). Hierarchal clustering analysis of the top 250 most significant DEGs, enriched for keratins and chemokines (Supplementary Table S6), also showed high intra-group similarity (Supplementary Figure S3).

Interrogation of DEG-level regulation revealed significant overlap in DEG lists between wound time points (compared to skin; Figure 3B). A total of 6,175 genes were upregulated in wounds (any time-point) compared to skin. Of these, 503 were specific to D1, 415 specific to D3, 238 specific to D7 and 1,670 specific to D14. Fewer genes were downregulated in wounds (4,288 at any time-point), with the highest number of genes again specific to the D14 comparison (980). To independently validate the RNA-Seq data, qRT-PCR was performed on a subset of upregulated and downregulated DEGs at each wound stage, which strongly correlated with RNA-Seq data (*R* > 0.94 and *p* < 0.001; Supplementary Figure S3).

Functional annotation was next performed on the full list of genes induced or repressed at each wound time-point, focusing on metallome-associated keywords and pathways (Figure 3C, Supplementary Table S7). At all wound time points, upregulated genes were highly annotated for calcium (UniProt KB: KW-0106), while enrichment for other metal-linked terms was wound stage specific. For example, in the D14 wound upregulated DEG group there was significant annotation for the terms metal-binding (UniProt KB: KW-0479; −Log_10_ *P*adjust = 5.08), zinc (UniProt KB: KW-0862; −Log_10_ *P*adjust = 2.11) and magnesium (UniProt KB: KW-0460; −Log_10_ *P*adjust = 1.38). Downregulated genes were instead highly enriched for oxidoreductase (UniProt KB: KW-0560), metal-binding, iron, and magnesium from D3 onwards. In summary, our newly generated wound RNA-Seq data show, for the first time, a close association between metals and wound repair at the global transcription level.

### Metal-Linked Genes Are Significantly Overrepresented in Murine and Human Wounds

In our RNA-Seq data set, 6,175 genes were upregulated upon injury (at any time-point), with 967 (15.7%) also present in the combined UniProt KB metal list (455 more genes than expected by chance, 1.89-fold overrepresentation, −log_10_ *p* = 94.3; Figure 3D). Hierarchal clustering of the 250 most significant of these overlapping genes revealed four distinct sub-clusters with unique transcriptional signatures, annotated to specific functional processes (Supplementary Figure S4; Supplementary Tables S8, S9). Ingenuity Pathway Analysis (IPA) identified key upstream regulators for each sub-cluster (Supplementary Figure S4; Supplementary Tables S10, S11). As a key identified process was ECM, we extracted subsets of ECM-linked genes from UniProt and compared their temporal expression (RNA-Seq data) to metal profile (Supplementary Figure S5).

Of the 4,288 downregulated genes, 684 (16%) were also in the combined UniProt KB metal list (329 more genes than expected by chance, 1.92-fold over-representation, −log_10_ *p* = 66.8; Figure 3D; Supplementary Table S12). Overrepresentation analysis was subsequently performed for wound upregulated DEGs for each individual metal (UniProt KB lists), displayed as a heat map (Figure 3E). Interestingly, manganese and calcium (particularly at D7) were the most significantly overrepresented metal-gene lists across healing, while zinc was overrepresented in wound DEGs only at D14 (Supplementary Table S13).

Due to the high overrepresentation of metal-linked genes in mouse wounds, we next interrogated a published human wound microarray data set comparing skin, acute wound (immediately after biopsy), and wounds at day 3 and 7 post-injury (GSE28914; Nuutila et al., 2012). Similar to the mouse data set, samples clustered by time point, with the highest similarity in expression profiles observed between D3 and D7 (Supplementary Figure S6). We then assessed metal gene enrichment (using the combined human UniProt KB metal lists), where of the genes that were upregulated in human wounds, 827 were also found in the combined metal gene list (398 more genes than expected by chance, 1.93-fold overrepresentation, −log_10_ *p* = 85.3; Figure 3F; Supplementary Table S14). DEGs downregulated in human wounds were also significantly enriched for metals (74 more genes than expected by chance, 2.12-fold overrepresentation, −log_10_ *p* = 17.4; Figure 3F). Additionally, we performed overrepresentation analysis on human wound-upregulated DEGs separated by wound time point and metal (Figure 3G; Supplementary Table S15). Taken together, these data clearly highlight metal-specific wound gene enrichment profiles that are temporally altered in mouse and human wounds.

### DEGs Cluster Into Wound-Relevant Subsets That Are Also Highly Enriched for Metals

**FIGURE 4:**
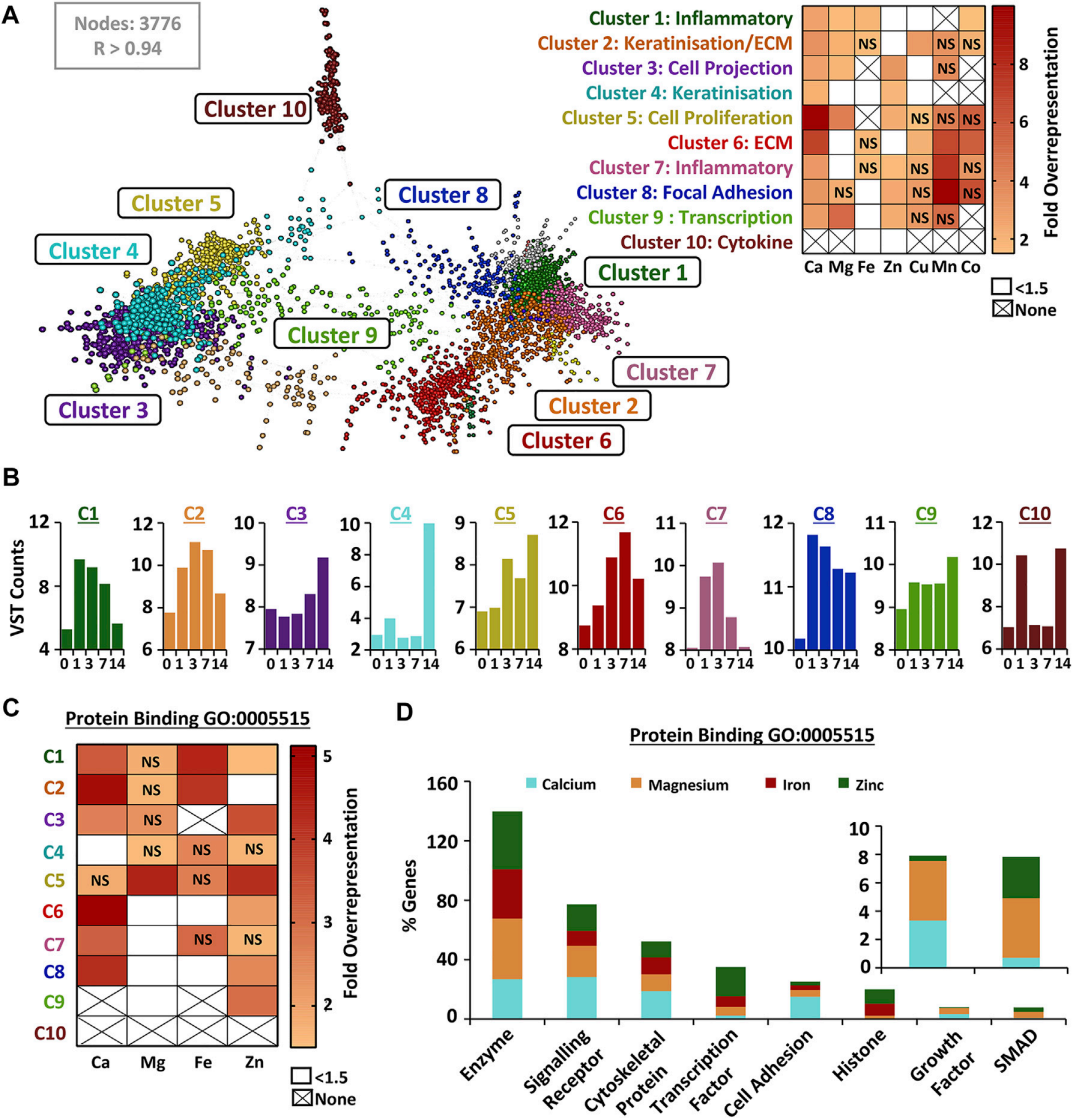
Wound-induced genes cluster by biological function and show process-specific metal overrepresentation. Wound upregulated differentially expressed genes (DEGs) were clustered **(A)** by similarity in expression signatures (VST data) using a Markov clustering algorithm. Clusters named based on functional annotation. Overrepresentation of UniProt Knowledge Base metal genes in defined clusters (heat map, right). Expression profile for each cluster shown in **(B)**. Overrepresentation performed on metal-linked genes from the protein binding gene ontology (GO) group (GO:0005515) in **(C)**, with specific subset contribution in **(D)**. NS, non-significant; None, no genes in metal group; VST, variance stabilised transformation; R, Pearson’s correlation.

Wound-upregulated DEGs from RNA-Seq analysis (combined list from all wound time points) were next independently clustered (Markov Cluster Algorithm) based on expression signature, and each cluster assigned a dominant wound-linked functional annotation (Figure 4A; Supplementary Table S16). The identified upregulated gene clusters were linked to inflammation, proliferation, keratinisation and ECM. Overrepresentation analysis, displayed as a heat map (Figure 4A), demonstrated specific and significant enrichment for different metal-linked gene groups (UniProt KB) across 9 out of 10 identified wound-upregulated DEG clusters (Supplementary Table S17). Normalised count data were then used to generate mean cluster expression profiles (Figure 4B). For example, genes in cluster four are overrepresented for both calcium and zinc, have a profile of expression that peaks at D14, and are associated with keratinisation. Hence, for each cluster we show linked function, metal association (Figure 4A) and expression profile (Figure 4B). Next, we extracted subsets of genes from the largest of the assessed UniProt metal lists (calcium, magnesium, iron and zinc) that were associated with protein binding (GO:0005515), transporter activity (GO:0005215) and signalling receptor activity (GO: 0038023). The protein binding GO group was the only group to feature a high proportion of genes from all four metal lists (Supplementary Table S18). Interestingly, cluster-specific overrepresentation was altered for metal protein binding genes versus all metal genes (Figure 4C; Supplementary Tables S18, S19). For example, calcium- and zinc-linked genes were no longer significantly overrepresented in cluster 4 (keratinisation) while zinc became overrepresented in cluster 6 (ECM) when focusing on protein binding subsets. Stepping down a level of GO annotation revealed that a high proportion of protein binding metal-linked genes were enzyme and signalling receptor binding (Figure 4D).

### Calcium Shows Temporal Wound Level Regulation That Converges on Key Time-Point Specific Upstream Regulators

Given their high representation in wound DEGs (Figures 3, 4), we explored calcium-specific genes (i.e., only genes found in the calcium UniProt KB group) within our murine RNA-Seq data set and the published human microarray data set in more detail. See Supplementary Figure S6 for volcano plots of calcium-specific changes in wound gene expression at each healing time-point (versus D0; Supplementary Table S20), with corresponding time-point dependent functional annotation (Supplementary Figure S7, Supplementary Tables S21, S22).

**FIGURE 5:**
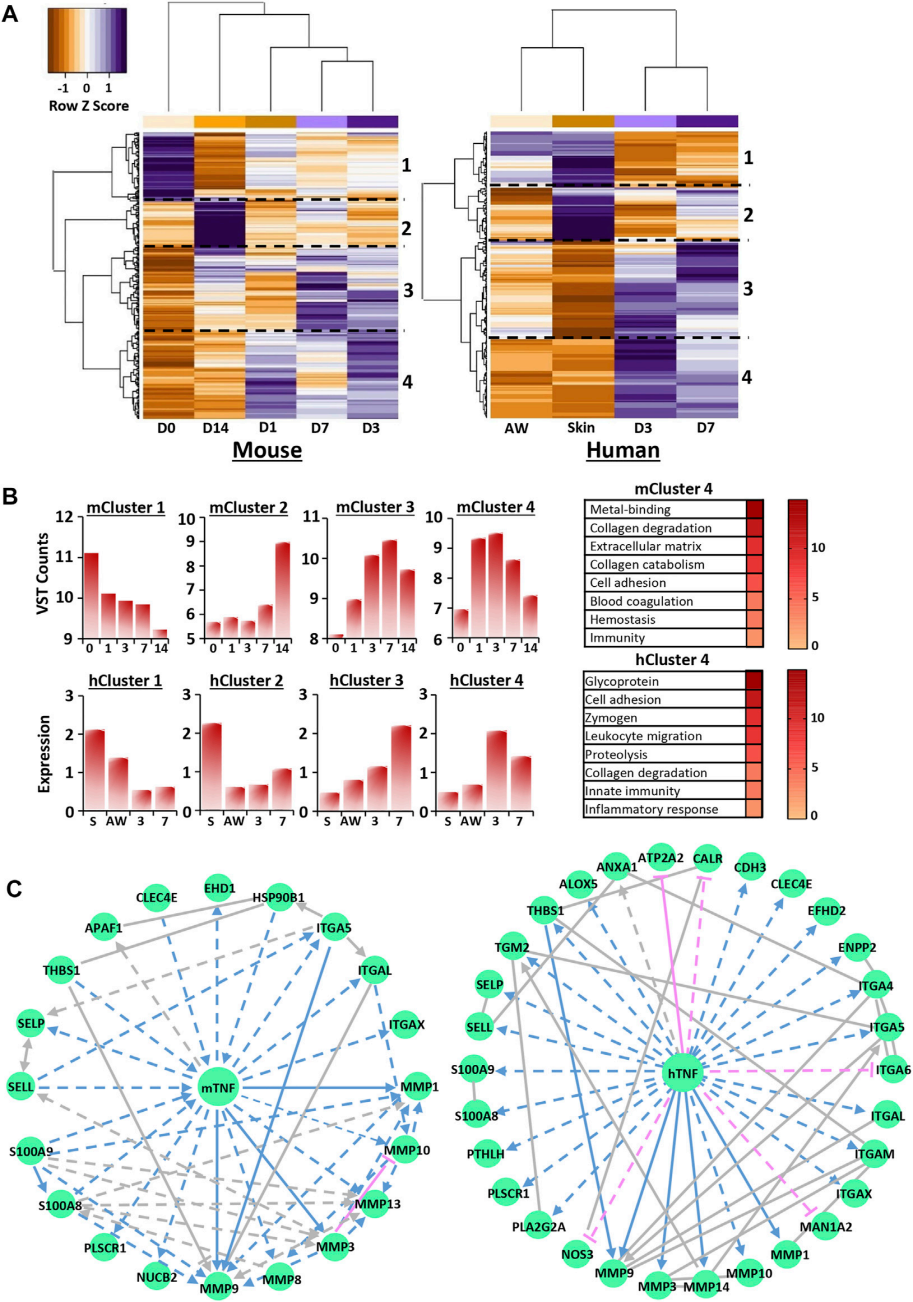
Transcriptional regulation of calcium is wound stage specific and converges on key upstream regulators. Hierarchal clustering of the top 250 most significant genes in mouse RNA-Seq and human microarray filtered from the calcium UniProt knowledge base (KB) list for each species [KW-0106; **(A)**]. D0 = normal skin. D1 = day 1 post-wounding. Cut-off = Benjamini-Hochberg adjusted *p* value (*P*adjust) < 0.05 and log_2_ fold change = 0.58. Functional annotation of identified clusters and their expression profiles **(B)**. VST = variance stabilised transformation. Network analysis identifying TNF as a top transcriptional regulator for cluster 4 **(C)**. m, mouse; h, human.

Focusing on the 250 most significant calcium-specific genes (filtered by fold change <1.5 and significance) in both mouse and human data sets, hierarchical clustering revealed greatest expression similarity between D3 and D7 wounds (Figure 5A, Supplementary Tables S23, S24). Note, D3 and D7 were also the time points where murine wound tissue calcium levels were strongly increased (ICP-MS; Figure 1A). Hierarchical clustering at the gene level revealed four distinct clusters for both heat maps (labelled 1–4; Figure 5A). Functional annotation analysis showed that calcium-linked genes in cluster 3 (high expression at D3 and D7 in mouse and human wounds) were strongly associated with a range of wound processes, including ECM (−log_10_ *p* = 18.5 in mouse; −log_10_ *p* = 6.8 in human) and cell adhesion (−log_10_ *p* = 4.9 in mouse; −log_10_ *p* = 5 in human) (Figure 5B; Supplementary Tables S25, S26). Cluster 4 (high expression at D1, D3 and D7 in mouse and D3 and D7 in human) contained calcium-linked genes involved in collagen degradation (−log_10_ *p* = 11.7 in mouse; −log_10_ *p* = 5 in human) and immunity (−log_10_ *p* = 2.5 in mouse; −log_10_ *p* = 8 in human).

Next, IPA analysis was performed on RNA-Seq and microarray data to determine putative upstream regulators for each calcium-specific cluster. Interestingly, multiple potential upstream regulators, common to both mouse and human, were identified for clusters 1, 3 and 4 (Supplementary Tables S27, S28). These included SMARCA4 (cluster 1), TGFB1, alpha catenin, VEGFA (cluster 3) and TNF, EGF and IL6 (cluster 4). TGFB1, the top identified upstream regulator for cluster 3 in both mouse (activation score: 3.562, *p* = 2.96E-08) and human (activation score: 2.531, *p* = 3.19E-05), was linked to gene networks that contained multiple ECM components and ECM remodeling enzymes. TNF, one of the top predicted activators for cluster 4 for both mouse (activation score: 4.2, *p* = 6.83E-06) and human (activation score: 3.7, *p* = 5.31E-08), was used to construct networks dominated by epidermal and immune-linked genes (Figure 5C; Supplementary Tables S29, S30). Collectively, these data reveal high transcriptional regulation of calcium in wounds, particularly at D3 and D7 post-injury, suggesting that calcium may have new unappreciated roles in later stages of healing.

### Metal-Led Transcriptomic Analysis Identifies TNF as an Important Regulator of Calcium-Induced Epidermal Differentiation

**FIGURE 6:**
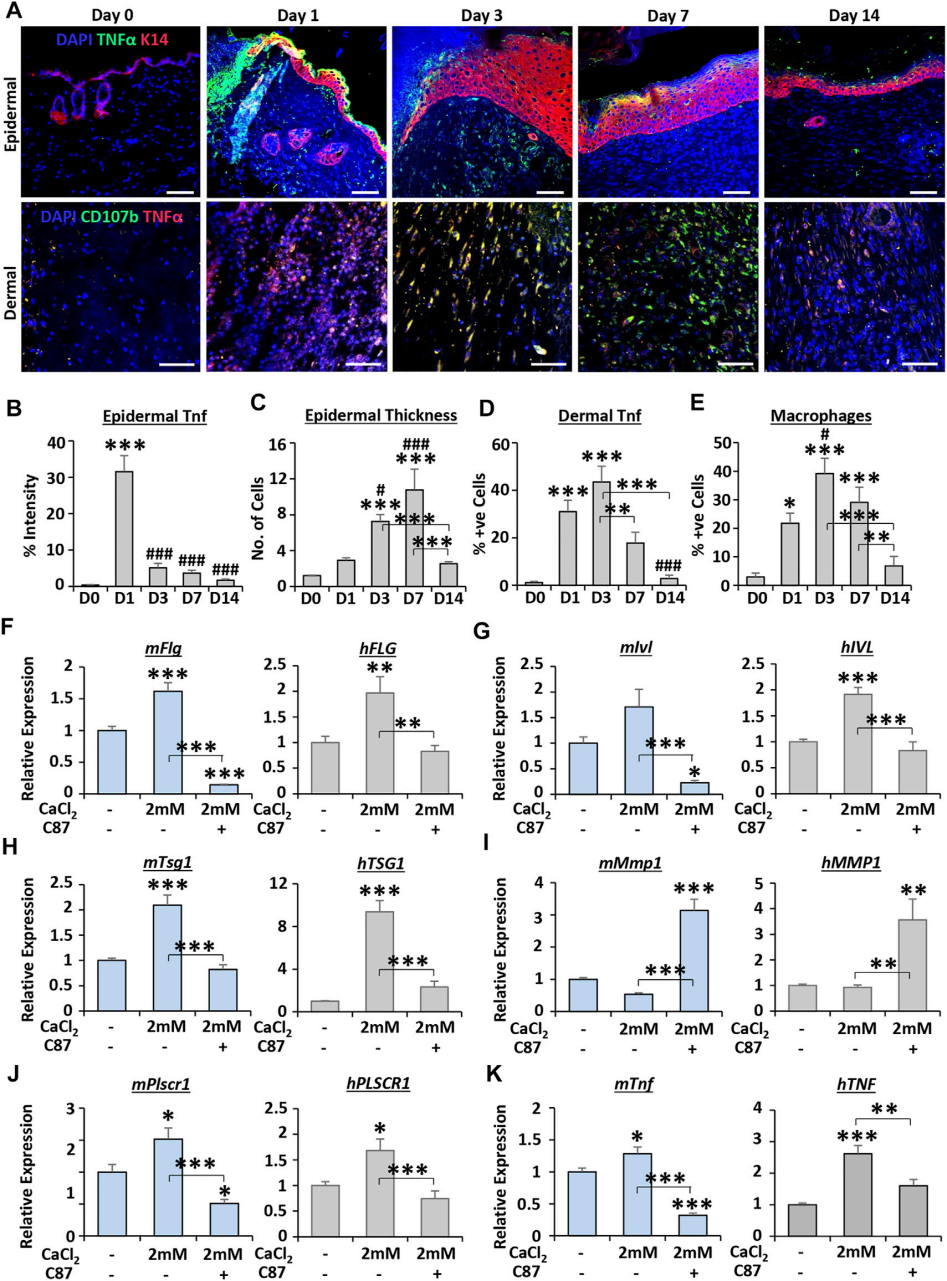
TNFα inhibitor treatment impairs calcium-dependent differentiation in keratinocytes. Mouse epidermal staining for keratin 14 (K14, red) and TNFα (green) and dermal staining for CD107b (green) and TNFα [red; **(A)**]. DAPI = blue nuclei. Bar = 50 µm. Quantification of epidermal TNFα **(B)**, epidermal thickness **(C)**, dermal TNFα **(D)** and macrophages [CD107b+ve **(E)**]. *n* = 5 mice. qRT-PCR for differentiation markers **(F–H)** and TNF-linked markers **(I–K)** in primary murine and human keratinocytes. C87 = TNFα inhibitor. *n* = 3 donors. Mean +SEM. One-way ANOVA with Tukey’s *post-hoc* analyses were performed. */# = *p* < 0.05, ** = *p* < 0.01 and ***/### = *p* < 0.001. Asterisk alone versus D0 or no calcium. Hashtag alone versus D1 in **(B–E)**.

Finally, we investigated the link between calcium and one of the top predicted upstream regulators (TNF) in more detail. We first performed immunofluorescence on mouse wound tissue to determine the temporal expression profile of TNFα in the epidermis and dermis throughout healing (Figure 6A). TNFα expression peaked at D1 in wound edge epidermis (*p* < 0.001; Figure 6B), while the thickness of the wound-edge neo-epidermis peaked at D7 (*p* < 0.001; Figure 6C). In the dermis, TNFα levels were significantly elevated at D1 and D3 versus D0 (both *p* < 0.001; Figure 6D), with expression correlating closely with macrophage numbers at these time points (Figure 6E).

We next focused on keratinocyte differentiation as a process of interest, based on our transcriptomic analysis, tissue staining, and the widely documented association between calcium and keratinocyte differentiation (Elias et al., 2002). Here, the influence of TNFα on calcium-induced keratinocyte differentiation was assessed in primary MEKs and HEKs. As expected, high calcium (2 mM CaCl_2_) significantly increased the expression of epidermal differentiation markers in mouse and human keratinocytes. However, this increase was strongly attenuated by co-treatment with the TNFα inhibitor, C87 (Figures 6F–H). Interestingly, treatment with C87 led to significant upregulation of *Mmp1*/*MMP1* (Figure 6I) and downregulation of *Plscr1*/*PLSCR1* (Figure 6J), genes predicted to be regulated by TNF in calcium cluster 4. The expression of *Tnf*/*TNF* itself was significantly increased following calcium treatment (*p* < 0.01—*p* < 0.001), an effect which was reversed by C87 (Figure 6K). Thus, these data suggest an important wound-relevant functional linkage between TNF and calcium in keratinocytes, validating our global transcriptional interrogation approach.

## Discussion

This study is the first to comprehensively evaluate changes in multiple endogenous metals across a murine healing time course and link these temporal metal fluctuations to the global wound transcriptome. Metallomics is an emerging, but still relatively understudied, area of biology. Recent reports have linked specific metals to pathologies, including ageing (Möller et al., 2019) and diabetes (Fernández-Cao et al., 2019). However, understanding of how these endogenous bio-metals contribute to normal function and disease states remains limited. In the skin, almost all metallome research to date has focussed on measuring metal bioaccumulation following treatment or occupational exposure (Lidén et al., 2008; Al-Dayel et al., 2011; Bianco et al., 2014), rather than matching endogenous metal levels to function in normal skin biology.

Data presented in the current study reveal metal-specific, time-dependent variations in calcium, magnesium, iron, zinc, aluminium, copper and manganese across murine skin healing. Crucially, each wound metal fluctuated in a manner that provides an indication into their role(s) in cellular aspects of healing. For example, ICP-MS-measured calcium levels peaked at D3 and D7 (the inflammatory and proliferative stages of healing). Note, the majority of existing wound literature focuses on measuring calcium flashes or waves induced within minutes of injury (Xu and Chisholm, 2011; Yoo et al., 2012; Razzell et al., 2013; Tu et al., 2019). A tissue calcium peak at D3/D7 is much more in line with the tissue-relevant roles for calcium in regulating immune cell function and cellular proliferation (Schwarz et al., 2006; Vashi et al., 2017). Intriguingly, we report a substantial reduction in tissue calcium levels during remodelling (D14), a stage characterised by a reduction in cell infiltration and the cessation of many calcium-dependent processes (e.g., MMP production, Singh et al., 2015). Indeed, calcium-specific genes upregulated in D14 wounds were not associated with any upstream regulators, proposing they have no obvious wound-relevant cellular functions.

Copper significantly accumulates at day 7 PW, in line with its reported role as an essential cofactor in angiogenesis (Urso and Maffia, 2015; Das et al., 2016), where high numbers of newly formed blood vessels are observed (Hattori et al., 2009). Copper is also an essential cofactor for lysyl oxidase (Lucero and Kagan, 2006), a collagen stabilising enzyme (Huang et al., 2019), suggesting a role in early granulation tissue maturation. The transition metals iron, aluminium and manganese were all elevated in the later stages of healing (D7 and D14 PW). This pattern of temporal induction fits with the ECM deposition and remodelling stages of wound repair. In fact, we recently demonstrated that wound tissue iron is required for ECM deposition and remodelling, and significantly perturbed in diabetic healing (Wilkinson et al., 2019b; 2019c). Moreover, Craven et al. (2001) showed that overexpression of manganese superoxide dismutase led to collagen accumulation, while Treiber et al. (2009) demonstrated that manganese promoted the contraction of collagen gel lattices, a proxy for *in vivo* wound closure.

Interestingly, we did not observe significant alterations in the overall abundance of zinc throughout skin healing, despite its crucial roles in many wound-associated processes (e.g., metalloproteinase activity, Caley et al., 2015). This could indicate that the high basal levels of zinc observed in skin are sufficient to support wound healing requirements. Our results remain in line with previous studies in rat wounds (Coger et al., 2019; Lansdown et al., 1999), but crucially highlight a caveat of measuring total metals in digested tissue samples without assessing the state of existence or intracellular localisation of metals. Indeed, our tissue extraction process and measurement method was not compatible with preserving free versus bound metals, but a number of approaches could be utilised to gain this valuable information. To spatially differentiate metals *in situ*, laser-ablation ICP-MS (Cruz-Alonso et al., 2019) or synchrotron radiation x-ray fluorescence (Zhang et al., 2018) can be used. Moreover, elemental analysis alone does not allow assessment of free versus bound metal but combining ICP-MS with sophisticated proteomic approaches would permit identification of metal-containing proteins (Wang et al., 2019). While technologically challenging, future studies should thus focus on characterising the spatial localisation and biological availability of metals in tissues.

It is currently unclear how observed murine changes in wound metals will translate into human healing. However, we note that basal metal levels are comparable between human and murine skin (unpublished data), with metal transport proteins generally conserved between both species (Danks, 1986). Our transcriptional analysis also reveals key parallels in metal-linked genes and functions in mice and humans. Furthermore, our data focussed on acute wounds, yet a major future consideration will be to understand how metals may be altered in/or contribute to chronic wound healing. Indeed, the influence of excessive iron to chronic wound inflammation has been assessed previously (Sindrilaru et al., 2011). Metal composition may also modulate bacterial presence and pathogenicity (Ma et al., 2015), highlighting potential microbiome-metallome interactions as an attractive avenue for further exploration.

We took a global transcriptional approach to investigate the relationship between metals and the transcriptome across murine (our RNA-Seq data) and human acute wound healing (published microarray). The RNA-Seq data alone provides an important new resource for the research community, as there are no comprehensive publicly available murine wound time course RNA-Seq data sets. Our detailed interrogation of wound DEGs delivers vital new insight into the global ties between the metallome and transcriptome, revealing numerous metal-linked genes that are induced or repressed in a temporal manner. Markov clustering of wound-induced genes independently confirmed that metal enrichment was dependent on biological function. For example, calcium was strongly linked to proliferation, while both calcium and zinc, metals widely associated with epidermal differentiation (Elias et al., 2002; Inoue et al., 2014), were linked to keratinisation. Focusing only on the metal-linked genes that were associated with protein binding provided further information into their relevance in key wound-linked biological processes. Indeed, these data highlight the crucial importance of investigating associations between metals and biological function but are limited by virtue of assessing only transcriptional changes, which do not always correlate with protein levels and function (Takemon et al., 2021). Proteomic approaches should therefore be incorporated in future studies to fully elucidate the links between metals and the biology of wound repair.

IPA network analysis identified the wound-associated transcription factor, TNF, important in mediating inflammation in early repair (Ritsu et al., 2017), as a primary upstream regulator for calcium-linked gene clusters in mouse and human wounds. Finally, we performed cell-based studies to confirm a putative role for TNFα in calcium-induced keratinocyte differentiation. Here we demonstrated that inhibition of TNFα attenuated calcium-induced differentiation in mouse and human keratinocytes. It is important to note that previous studies show a role for TNFα in negatively augmenting the epidermal barrier in skin pathology (Basile et al., 2001; Marble et al., 2007; Danso et al., 2014). However, to our knowledge, this is the first time endogenous TNFα has been directly linked to calcium-induced epidermal differentiation in a normal reparative context in mice and humans.

## Conclusion

**FIGURE 7:**
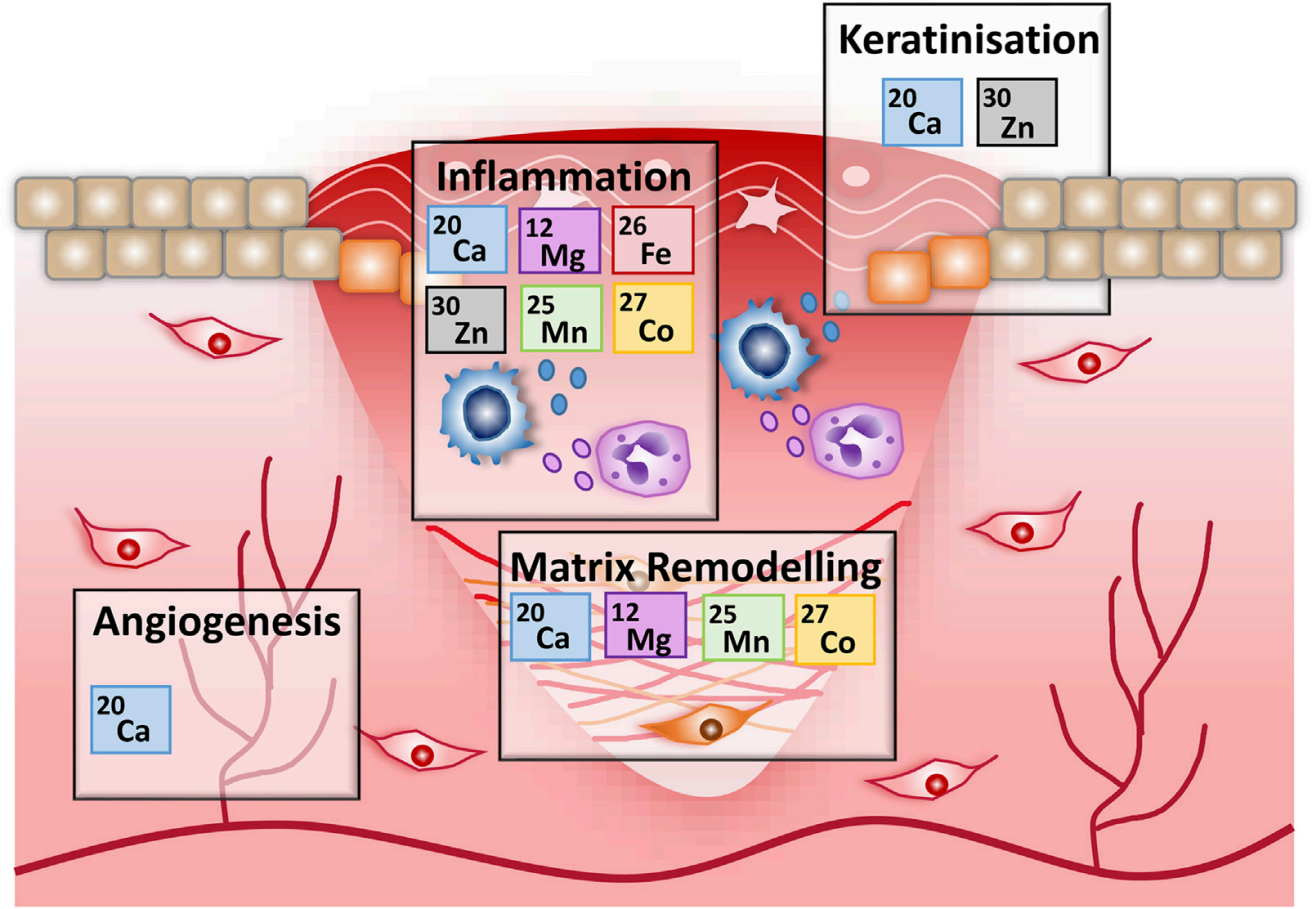
An overview of the potential roles for metals in wound repair. Schematic outlining the key metal associations identified in this work, for specific wound healing stages. Associations are based on metal gene list overrepresentation and functional annotation analysis of genes differentially expressed during murine wound healing.

In summary, our data provide the first comprehensive documentation of the metallomic/transcriptomic landscape of a healing wound, suggesting exciting new roles for metals in skin repair (overview provided in Figure 7). We note that previous studies have evaluated only a single metal (iron) in the context of healing pathology; which was shown to be perturbed in both diabetic murine and human wounds (Sindrilaru et al., 2011; Wilkinson et al., 2019c). Thus, our study not only reveals previously unappreciated and potentially wide-reaching roles for metals in wound repair, it also paves-the-way for essential follow on work to explore the cellular consequences of metallome dysbiosis during injury and disease in skin and other tissues.

## Data Availability

The data that support the findings of this study are available as Supplementary Material or from the corresponding author upon reasonable request. RNA-Seq data are deposited in the Sequence Read Archive database (https://www.ncbi.nlm.nih.gov/sra) under BioProject accession number PRJNA686364.

## References

[B1] Al-Dayel O. Hefne J. Al-Ajyan T. (2011). Human Exposure to Heavy Metals from Cosmetics. Orient. J. Chem. 27, 1–11.

[B2] Andreini C. Bertini I. Cavallaro G. Holliday G. L. Thornton J. M. (2008). Metal Ions in Biological Catalysis: from Enzyme Databases to General Principles. J. Biol. Inorg. Chem. 13, 1205–1218. 10.1007/s00775-008-0404-5 18604568

[B3] Basile J. R. Zacny V. Münger K. (2001). The Cytokines Tumor Necrosis Factor-α (TNF-α) and TNF-Related Apoptosis-Inducing Ligand Differentially Modulate Proliferation and Apoptotic Pathways in Human Keratinocytes Expressing the Human Papillomavirus-16 E7 Oncoprotein. J. Biol. Chem. 276, 22522–22528. 10.1074/jbc.M010505200 11306566

[B4] Berridge M. J. (1995). Calcium Signalling and Cell Proliferation. Bioessays 17, 491–500. 10.1002/bies.950170605 7575490

[B5] Bianco C. Adami G. Crosera M. Larese F. Casarin S. Castagnoli C. (2014). Silver Percutaneous Absorption after Exposure to Silver Nanoparticles: a Comparison Study of Three Human Skin Graft Samples Used for Clinical Applications. Burns 40, 1390–1396. 10.1016/j.burns.2014.02.003 24698780

[B6] Blighe K. (2019). EnhancedVolcano: Publication-Ready Volcano Plots with Enhanced Colouring and Labelling. Available at: https://github.com/kevinblighe/EnhancedVolcano (Accessed July 23, 2020).

[B7] Caley M. P. Martins V. L. C. O'Toole E. A. (2015). Metalloproteinases and Wound Healing. Adv. Wound Care 4, 225–234. 10.1089/wound.2014.0581 PMC4397992 25945285

[B8] Coger V. Million N. Rehbock C. Sures B. Nachev M. Barcikowski S. (2019). Tissue Concentrations of Zinc, Iron, Copper, and Magnesium during the Phases of Full Thickness Wound Healing in a Rodent Model. Biol. Trace Elem. Res. 191, 167–176. 10.1007/s12011-018-1600-y 30552609 PMC6656798

[B9] Craven P. A. Phillips S. L. Melhem M. F. Liachenko J. DeRubertis F. R. (2001). Overexpression of Manganese Superoxide Dismutase Suppresses Increases in Collagen Accumulation Induced by Culture of Mesangial Cells in High-media Glucose. Metabolism 50, 1043–1048. 10.1053/meta.2001.25802 11555836

[B10] Cruz-Alonso M. Fernandez B. Navarro A. Junceda S. Astudillo A. Pereiro R. (2019). Laser Ablation ICP-MS for Simultaneous Quantitative Imaging of Iron and Ferroportin in hippocampus of Human Brain Tissues with Alzheimer's Disease. Talanta 197, 413–421. 10.1016/j.talanta.2019.01.056 30771955

[B11] Danks D. M. (1986). Of Mice and Men, Metals and Mutations. J. Med. Genet. 23, 99–106. 10.1136/jmg.23.2.99 3519972 PMC1049562

[B12] Danso M. O. Van Drongelen V. Mulder A. Van Esch J. Scott H. Van Smeden J. (2014). TNF-α and Th2 Cytokines Induce Atopic Dermatitis-like Features on Epidermal Differentiation Proteins and Stratum Corneum Lipids in Human Skin Equivalents. J. Invest. Dermatol. 134, 1941–1950. 10.1038/jid.2014.83 24518171

[B13] Das A. Sudhahar V. Chen G.-F. Kim H. W. Youn S.-W. Finney L. (2016). Endothelial Antioxidant-1: a Key Mediator of Copper-dependent Wound Healing *In Vivo* . Sci. Rep. 6, 1–16. 10.1038/srep33783 27666810 PMC5036036

[B14] Elaïb Z. Adam F. Berrou E. Bordet J.-C. Prévost N. Bobe R. (2016). Full Activation of Mouse Platelets Requires ADP Secretion Regulated by SERCA3 ATPase-dependent Calcium Stores. Blood 128, 1129–1138. 10.1182/blood-2015-10-678383 27301859

[B15] Elias P. M. Ahn S. K. Denda M. Brown B. E. Crumrine D. Kimutai L. K. (2002). Modulations in Epidermal Calcium Regulate the Expression of Differentiation-specific Markers. J. Invest. Dermatol. 119, 1128–1136. 10.1046/j.1523-1747.2002.19512.x 12445203

[B16] Fernández-Cao J. C. Warthon-Medina M. H. Moran V. Arija V. Doepking C. Serra-Majem L. (2019). Zinc Intake and Status and Risk of Type 2 Diabetes Mellitus: a Systematic Review and Meta-Analysis. Nutrients 11, 1027. 10.3390/nu11051027 PMC6567047 31071930

[B17] Frykberg R. G. Banks J. (2015). Challenges in the Treatment of Chronic Wounds. Adv. Wound Care 4, 560–582. 10.1089/wound.2015.0635 PMC4528992 26339534

[B18] Graham R. M. Chua A. C. Herbison C. E. Olynyk J. K. Trinder D. (2007). Liver Iron Transport. Wjg 13, 4725. 10.3748/wjg.v13.i35.4725 17729394 PMC4611194

[B19] Greaves N. S. Ashcroft K. J. Baguneid M. Bayat A. (2013). Current Understanding of Molecular and Cellular Mechanisms in Fibroplasia and Angiogenesis during Acute Wound Healing. J. Dermatol. Sci. 72, 206–217. 10.1016/j.jdermsci.2013.07.008 23958517

[B20] Haldar M. Kohyama M. So A. Y.-L. Kc W. Wu X. Briseño C. G. (2014). Heme-mediated SPI-C Induction Promotes Monocyte Differentiation into Iron-Recycling Macrophages. Cell 156, 1223–1234. 10.1016/j.cell.2014.01.069 24630724 PMC4010949

[B21] Haraguchi H. (2017). Metallomics: the History over the Last Decade and a Future Outlook. Metallomics 9, 1001–1013. 10.1039/c7mt00023e 28758652

[B22] Hattori N. Mochizuki S. Kishi K. Nakajima T. Takaishi H. D'Armiento J. (2009). MMP-13 Plays a Role in Keratinocyte Migration, Angiogenesis, and Contraction in Mouse Skin Wound Healing. Am. J. Pathol. 175, 533–546. 10.2353/ajpath.2009.081080 19590036 PMC2716954

[B23] Heberle H. Meirelles G. V. da Silva F. R. Telles G. P. Minghim R. (2015). InteractiVenn: a Web-Based Tool for the Analysis of Sets through Venn Diagrams. BMC Bioinformatics 16, 1–7. 10.1186/s12859-015-0611-3 25994840 PMC4455604

[B24] Huang D. W. Sherman B. T. Lempicki R. A. (2009). Systematic and Integrative Analysis of Large Gene Lists Using DAVID Bioinformatics Resources. Nat. Protoc. 4, 44–57. 10.1038/nprot.2008.211 19131956

[B25] Huang M. Liu Z. Baugh L. DeFuria J. Maione A. Smith A. (2019). Lysyl Oxidase Enzymes Mediate TGF-Β1-Induced Fibrotic Phenotypes in Human Skin-like Tissues. Lab. Invest. 99, 514–527. 10.1038/s41374-018-0159-8 30568176

[B26] Inoue Y. Hasegawa S. Ban S. Yamada T. Date Y. Mizutani H. (2014). ZIP2 Protein, a Zinc Transporter, Is Associated with Keratinocyte Differentiation. J. Biol. Chem. 289, 21451–21462. 10.1074/jbc.M114.560821 24936057 PMC4118108

[B27] Lee S. Bi X. Reed R. B. Ranville J. F. Herckes P. Westerhoff P. (2014). Nanoparticle Size Detection Limits by Single Particle ICP-MS for 40 Elements. Environ. Sci. Technol. 48, 10291–10300. 10.1021/es502422v 25122540

[B28] Lidén C. Skare L. Nise G. Vahter M. (2008). Deposition of Nickel, Chromium, and Cobalt on the Skin in Some Occupations - Assessment by Acid Wipe Sampling. Contact Dermatitis 58, 347–354. 10.1111/j.1600-0536.2008.01326.x 18503684

[B29] Love M. I. Huber W. Anders S. (2014). Moderated Estimation of Fold Change and Dispersion for RNA-Seq Data with DESeq2. Genome Biol. 15, 550. 10.1186/s13059-014-0550-8 25516281 PMC4302049

[B30] Lucero H. A. Kagan H. M. (2006). Lysyl Oxidase: an Oxidative Enzyme and Effector of Cell Function. Cell. Mol. Life Sci. 63, 2304–2316. 10.1007/s00018-006-6149-9 16909208 PMC11136443

[B31] Ma L. Terwilliger A. Maresso A. W. (2015). Iron and Zinc Exploitation during Bacterial Pathogenesis. Metallomics 7, 1541–1554. 10.1039/c5mt00170f 26497057 PMC4836889

[B32] Mandeville J. T. H. Maxfield F. R. (1997). Effects of Buffering Intracellular Free Calcium on Neutrophil Migration through Three-Dimensional Matrices. J. Cell. Physiol. 171, 168–178. 10.1002/(sici)1097-4652(199705)171:2<168:aid-jcp7>3.0.co;2-m 9130464

[B33] Marble D. J. Gordon K. B. Nickoloff B. J. (2007). Targeting TNFα Rapidly Reduces Density of Dendritic Cells and Macrophages in Psoriatic Plaques with Restoration of Epidermal Keratinocyte Differentiation. J. Dermatol. Sci. 48, 87–101. 10.1016/j.jdermsci.2007.06.006 17689932 PMC2703191

[B34] Möller H. E. Bossoni L. Connor J. R. Crichton R. R. Does M. D. Ward R. J. (2019). Iron, Myelin, and the Brain: Neuroimaging Meets Neurobiology. Trends Neurosciences 42, 384–401. 10.1016/j.tins.2019.03.009 31047721

[B35] Nirmal A. J. Regan T. Shih B. B. Hume D. A. Sims A. H. Freeman T. C. (2018). Immune Cell Gene Signatures for Profiling the Microenvironment of Solid Tumors. Cancer Immunol. Res. 6, 1388–1400. 10.1158/2326-6066.CIR-18-0342 30266715

[B36] Nuutila K. Siltanen A. Peura M. Bizik J. Kaartinen I. Kuokkanen H. (2012). Human Skin Transcriptome during Superficial Cutaneous Wound Healing. Wound Repair Regen. 20, 830–839. 10.1111/j.1524-475X.2012.00831.x 23082929

[B37] Plaisier S. B. Taschereau R. Wong J. A. Graeber T. G. (2010). Rank-rank Hypergeometric Overlap: Identification of Statistically Significant Overlap between Gene-Expression Signatures. Nucleic Acids Res. 38, e169. 10.1093/nar/gkq636 20660011 PMC2943622

[B38] Pretorius E. Bester J. Vermeulen N. Lipinski B. (2013). Oxidation Inhibits Iron-Induced Blood Coagulation. Curr. Drug Targets 14, 13–19. 10.2174/138945011131401000310.2174/138945013804806541 23170793 PMC3580830

[B39] R Core Team (2020). A Language and Environment for Statistical Computing. Vienna, Australia: R Foundation for Statistical Computing. Available at: https://www.R-project.org/ (Accessed July 23, 2020).

[B40] Razzell W. Evans I. R. Martin P. Wood W. (2013). Calcium Flashes Orchestrate the Wound Inflammatory Response through DUOX Activation and Hydrogen Peroxide Release. Curr. Biol. 23, 424–429. 10.1016/j.cub.2013.01.058 23394834 PMC3629559

[B41] Ritsu M. Kawakami K. Kanno E. Tanno H. Ishii K. Imai Y. (2017). Critical Role of Tumor Necrosis Factor-α in the Early Process of Wound Healing in Skin. J. Dermatol. Dermatol. Surg. 21, 14–19. 10.1016/j.jdds.2016.09.001

[B42] Schwarz E. C. Wissenbach U. Niemeyer B. A. Strauß B. Philipp S. E. Flockerzi V. (2006). TRPV6 Potentiates Calcium-dependent Cell Proliferation. Cell Calcium 39, 163–173. 10.1016/j.ceca.2005.10.006 16356545

[B43] Sindrilaru A. Peters T. Wieschalka S. Baican C. Baican A. Peter H. (2011). An Unrestrained Proinflammatory M1 Macrophage Population Induced by Iron Impairs Wound Healing in Humans and Mice. J. Clin. Invest. 121, 985–997. 10.1172/JCI44490 21317534 PMC3049372

[B44] Singh D. Srivastava S. K. Chaudhuri T. K. Upadhyay G. (2015). Multifaceted Role of Matrix Metalloproteinases (MMPs). Front. Mol. Biosci. 2, 19. 10.3389/fmolb.2015.00019 25988186 PMC4429632

[B45] Singh V. Verma K. (2018). Metals from Cell to Environment: Connecting Metallomics with Other Omics. Open. J. Plant Sci. 3, 1–14.

[B46] Söllner J. F. Leparc G. Hildebrandt T. Klein H. Thomas L. Stupka E. (2017). An RNA-Seq Atlas of Gene Expression in Mouse and Rat normal Tissues. Sci. Data 4, 170185. 10.1038/sdata.2017.185 29231921 PMC5726313

[B47] Takemon Y. Chick J. M. Gerdes Gyuricza I. Skelly D. A. Devuyst O. Gygi S. P. (2021). Proteomic and Transcriptomic Profiling Reveal Different Aspects of Aging in the Kidney. Elife 10, e62585. 10.7554/eLife.62585 33687326 PMC8096428

[B48] Treiber N. Peters T. Sindrilaru A. Huber R. Kohn M. Menke A. (2009). Overexpression of Manganese Superoxide Dismutase in Human Dermal Fibroblasts Enhances the Contraction of Free Floating Collagen Lattice: Implications for Ageing and Hyperplastic Scar Formation. Arch. Dermatol. Res. 301, 273–287. 10.1007/s00403-009-0935-9 19306099

[B49] Tu C.-L. Celli A. Mauro T. Chang W. (2019). Calcium-sensing Receptor Regulates Epidermal Intracellular Ca2+ Signaling and Re-epithelialization after Wounding. J. Invest. Dermatol. 139, 919–929. 10.1016/j.jid.2018.09.033 30404020 PMC6431556

[B50] Urso E. Maffia M. (2015). Behind the Link between Copper and Angiogenesis: Established Mechanisms and an Overview on the Role of Vascular Copper Transport Systems. J. Vasc. Res. 52, 172–196. 10.1159/000438485 26484858

[B51] Vashi N. Andrabi S. B. A. Ghanwat S. Suar M. Kumar D. (2017). Ca2+-dependent Focal Exocytosis of Golgi-Derived Vesicles Helps Phagocytic Uptake in Macrophages. J. Biol. Chem. 292, 5144–5165. 10.1074/jbc.M116.743047 28174296 PMC5392664

[B52] Wang H. Yan A. Liu Z. Yang X. Xu Z. Wang Y. (2019). Deciphering Molecular Mechanism of Silver by Integrated Omic Approaches Enables Enhancing its Antimicrobial Efficacy in *E. coli* . Plos Biol. 17, e3000292. 10.1371/journal.pbio.3000292 31181061 PMC6557469

[B53] Warnes G. R. Bolker B. Bonebakker L. Gentleman R. Huber W. Liaw A. (2020). Gplots: Various R Programming Tools for Plotting Data. Available at: https://CRAN.R-project.org/package=gplots (Accessed July 23, 2020).

[B54] Wei C. Wang X. Chen M. Ouyang K. Song L.-S. Cheng H. (2009). Calcium Flickers Steer Cell Migration. Nature 457, 901–905. 10.1038/nature07577 19118385 PMC3505761

[B55] Wilkinson H. N. Clowes C. Banyard K. L. Matteuci P. Mace K. A. Hardman M. J. (2019a). Elevated Local Senescence in Diabetic Wound Healing Is Linked to Pathological Repair via CXCR2. J. Invest. Dermatol. 139, 1171–1181. 10.1016/j.jid.2019.01.005 30684552

[B56] Wilkinson H. N. Hardman M. J. (2020). Senescence in Wound Repair: Emerging Strategies to Target Chronic Healing Wounds. Front. Cell Dev. Biol. 8, 773. 10.3389/fcell.2020.00773 32850866 PMC7431694

[B57] WilkinsonH. N.RobertsE. R.StaffordA. R.BanyardK. L.MatteucciP.MaceK. A. (2019b). Tissue Iron Promotes Wound Repair via M2 Macrophage Polarization and the Chemokine (C-C Motif) Ligands 17 and 22. Am. J. Pathol. 189, 2196–2208. 10.1016/j.ajpath.2019.07.015 31465751

[B58] Wilkinson H. N. Upson S. E. Banyard K. L. Knight R. Mace K. A. Hardman M. J. (2019c). Reduced Iron in Diabetic Wounds: An Oxidative Stress-dependent Role for STEAP3 in Extracellular Matrix Deposition and Remodeling. J. Invest. Dermatol. 139, 2368–2377. 10.1016/j.jid.2019.05.014 31176711

[B59] Xu S. Chisholm A. D. (2011). A Gαq-Ca2+ Signaling Pathway Promotes Actin-Mediated Epidermal Wound Closure in *C. elegans* . Curr. Biol. 21, 1960–1967. 10.1016/j.cub.2011.10.050 22100061 PMC3237753

[B60] Yannone S. M. Hartung S. Menon A. L. Adams M. W. Tainer J. A. (2012). Metals in Biology: Defining Metalloproteomes. Curr. Opin. Biotechnol. 23, 89–95. 10.1016/j.copbio.2011.11.005 22138493 PMC3273585

[B61] Yoo S. K. Freisinger C. M. LeBert D. C. Huttenlocher A. (2012). Early Redox, Src Family Kinase, and Calcium Signaling Integrate Wound Responses and Tissue Regeneration in Zebrafish. J. Cell. Biol. 199, 225–234. 10.1083/jcb.201203154 23045550 PMC3471241

[B62] Zhang R. Li L. Sultanbawa Y. Xu Z. P. (2018). X-ray Fluorescence Imaging of Metals and Metalloids in Biological Systems. Am. J. Nucl. Med. Mol. Imaging 8, 169–188. 30042869 PMC6056246

[B63] Zhu A. Ibrahim J. G. Love M. I. (2019). Heavy-tailed Prior Distributions for Sequence Count Data: Removing the Noise and Preserving Large Differences. Bioinformatics 35, 2084–2092. 10.1093/bioinformatics/bty895 30395178 PMC6581436

