# Supplementary figures and images for: Combined Metallomics/Transcriptomics Profiling Reveals a Major Role for Metals in Wound Repair
Source: Front Cell Dev Biol. 2021 Nov 30;9:788596. doi: 10.3389/fcell.2021.788596 (PMC8669724; doi:10.3389/fcell.2021.788596)

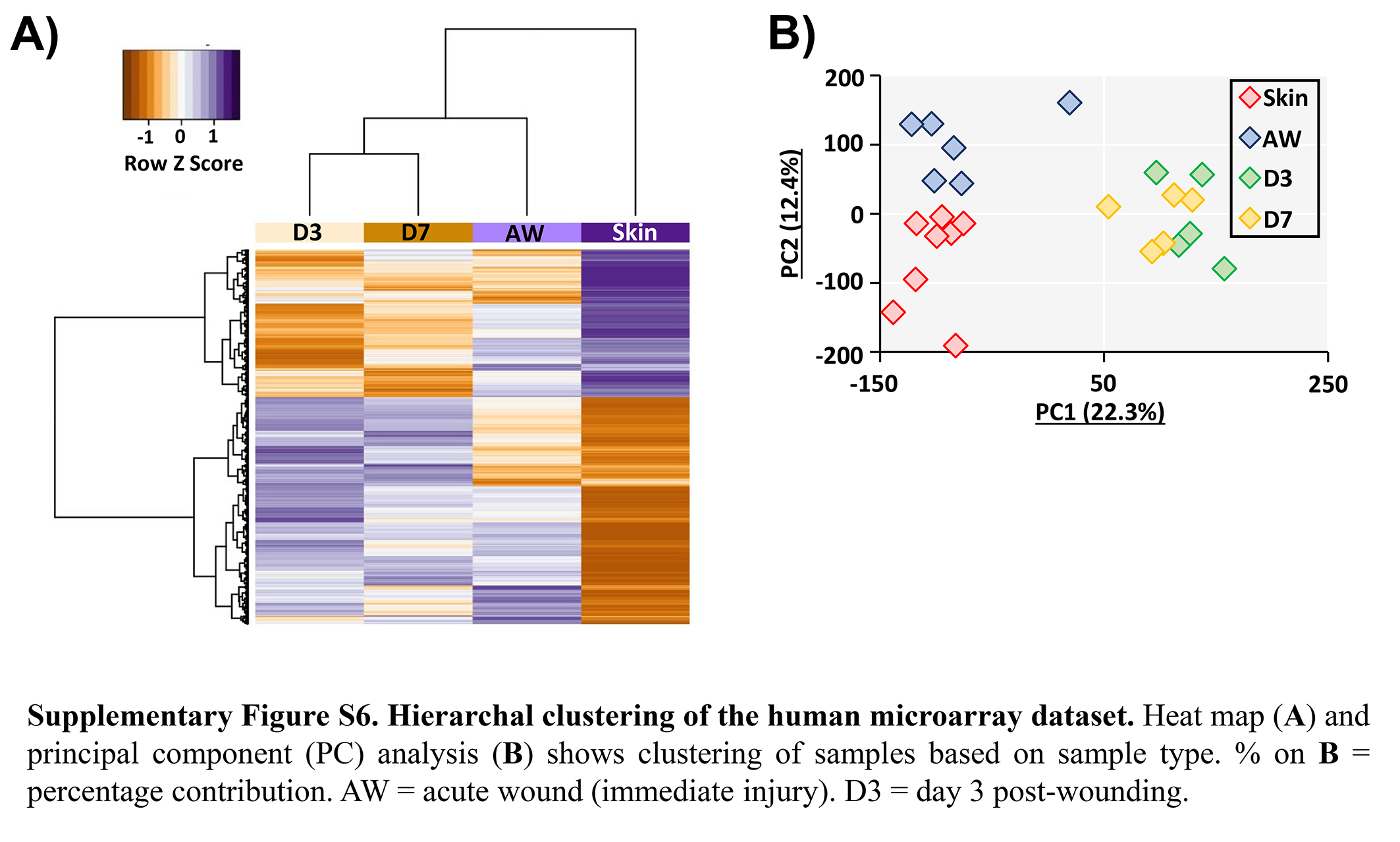

Supplement: Supplementary file 1 [file Image6.TIF]

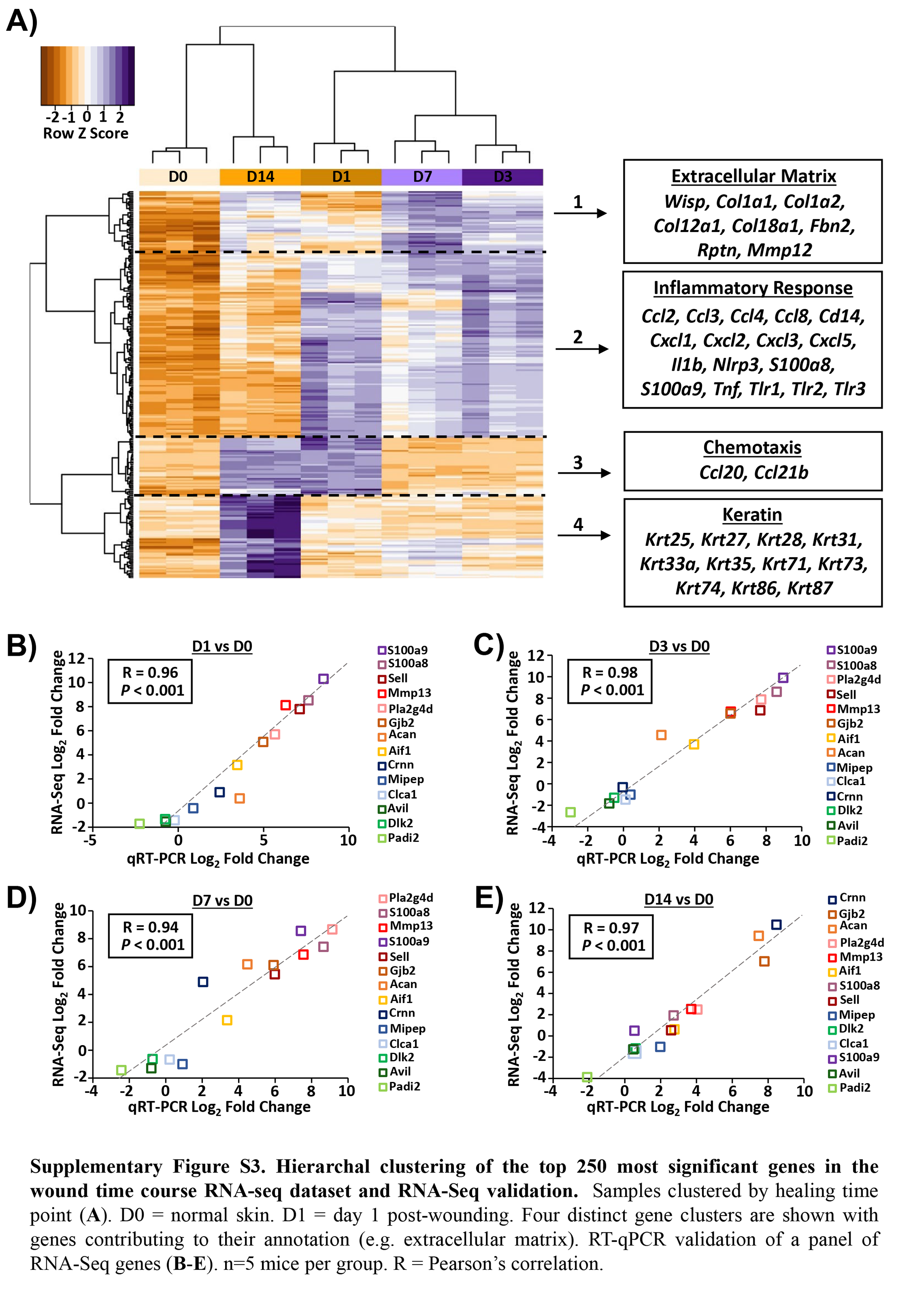

Supplement: Supplementary file 2 [file Image3.TIF]

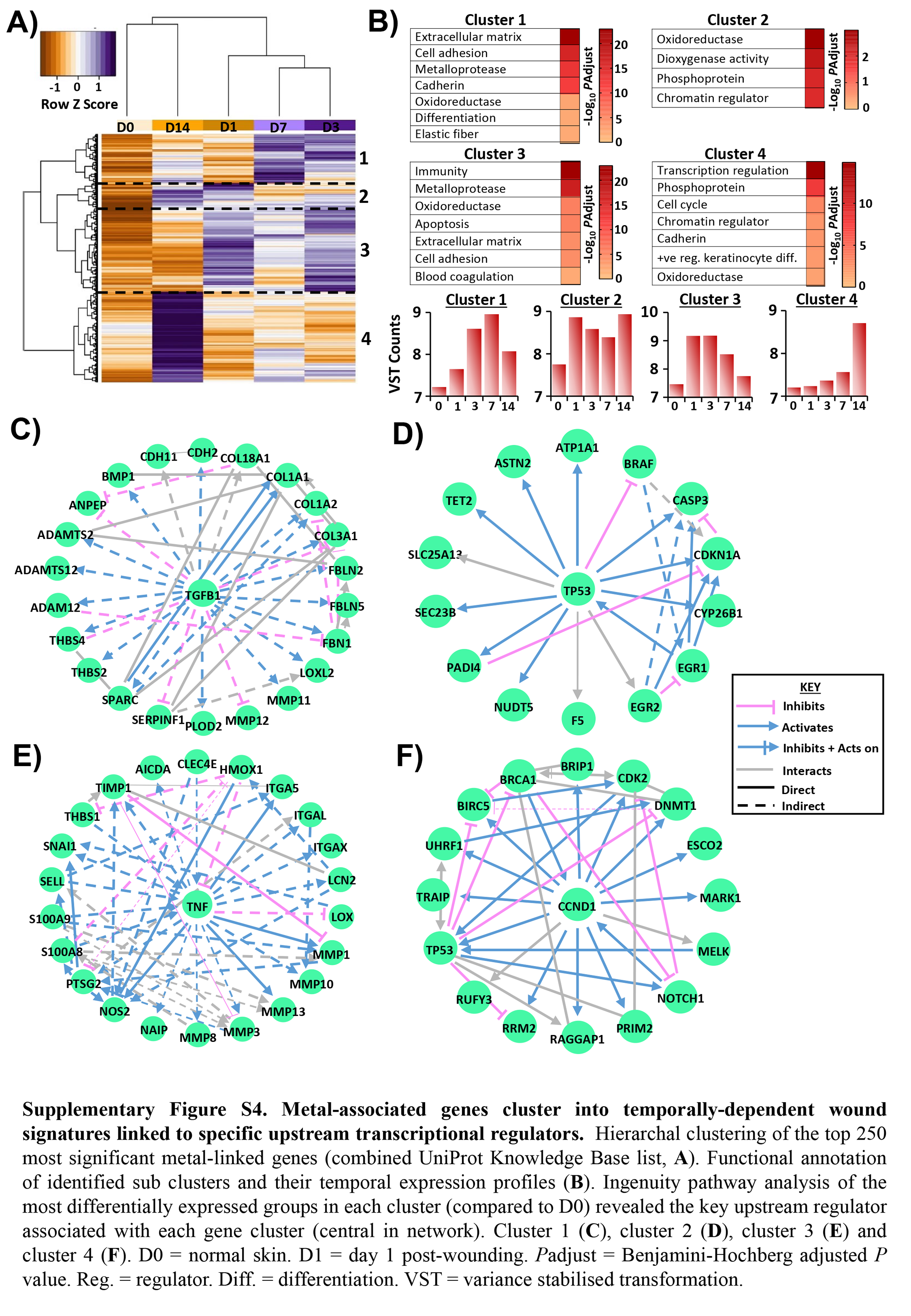

Supplement: Supplementary file 3 [file Image4.TIF]

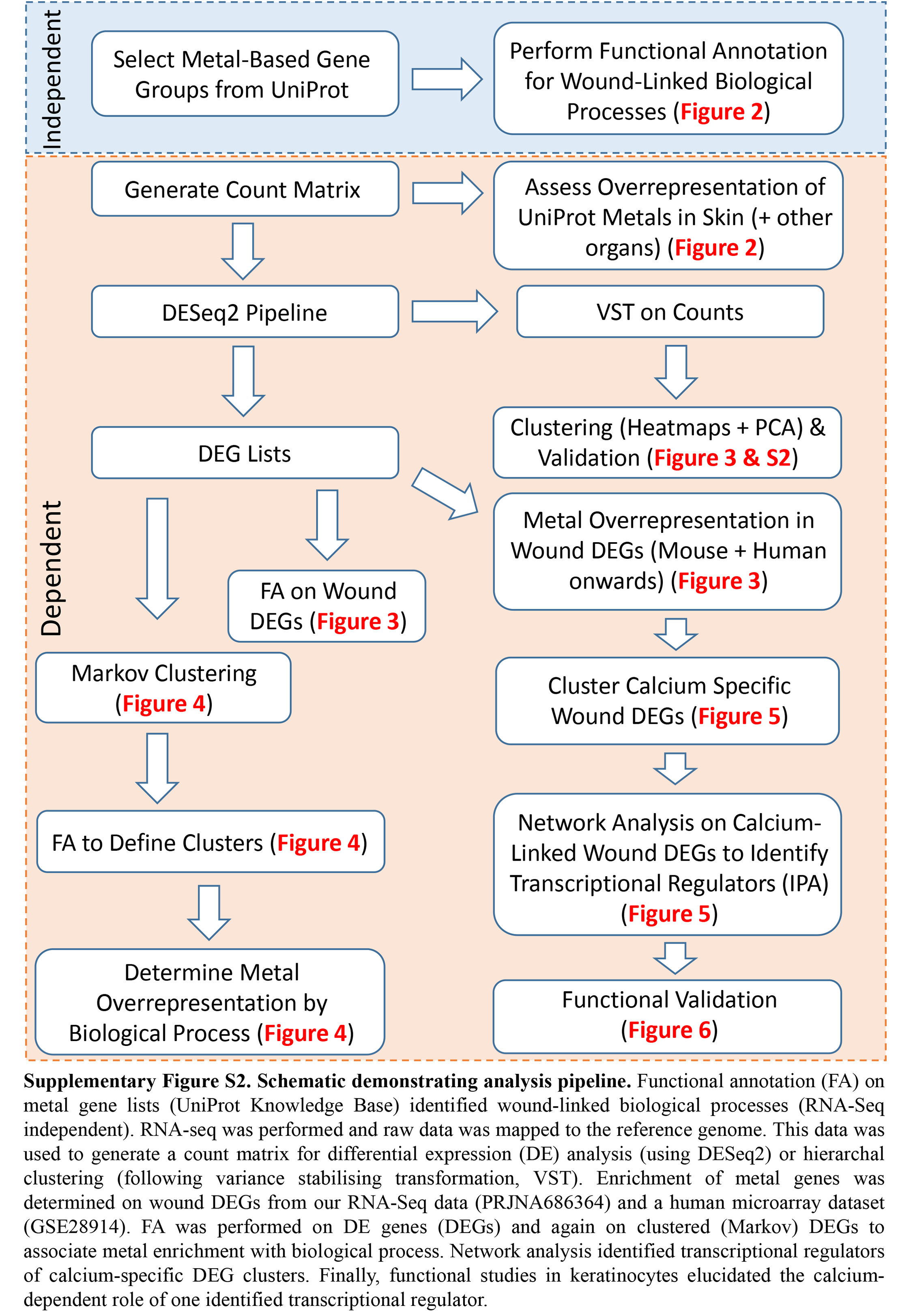

Supplement: Supplementary file 4 [file Image2.tif]

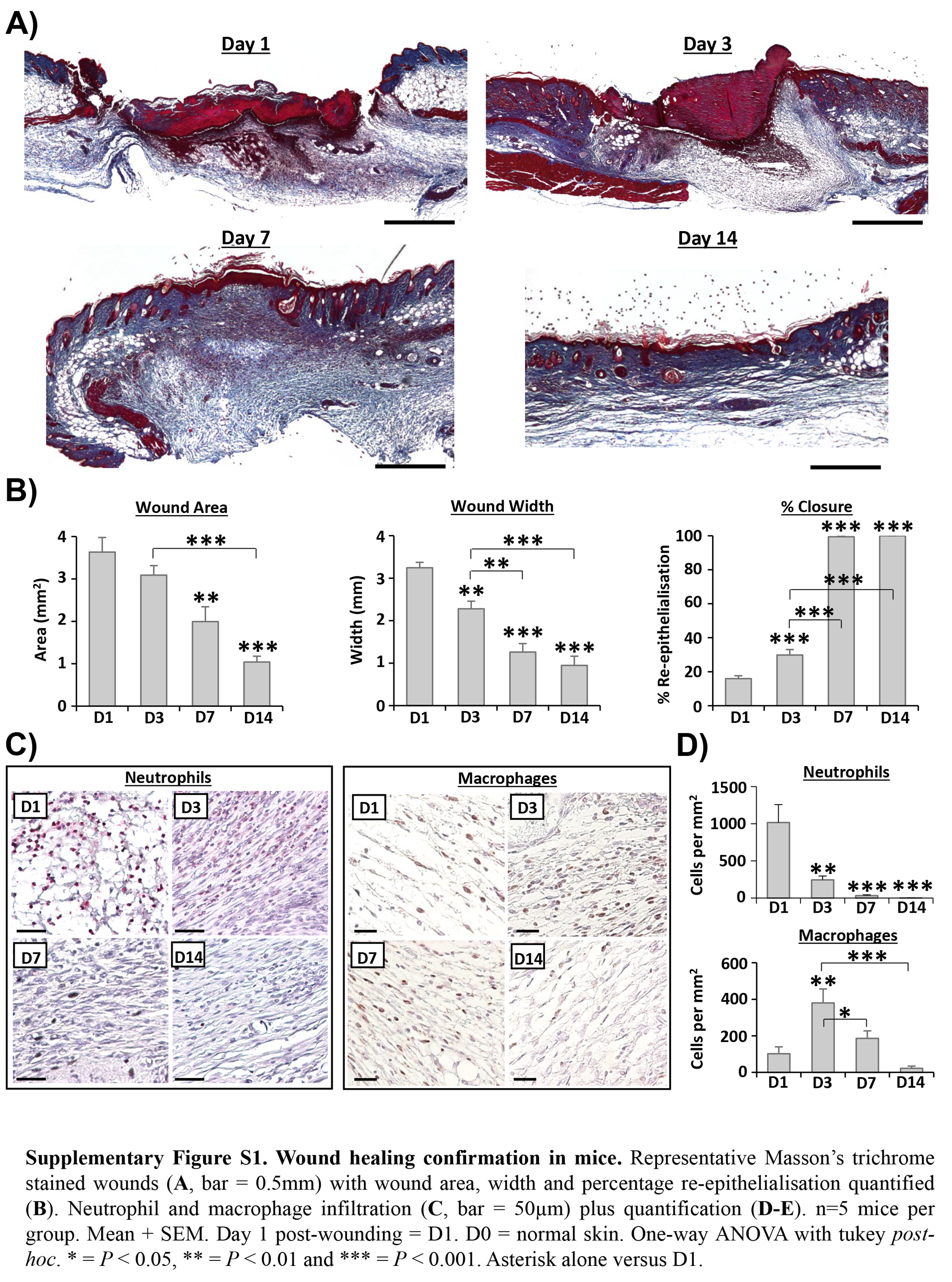

Supplement: Supplementary file 5 [file Image1.TIF]

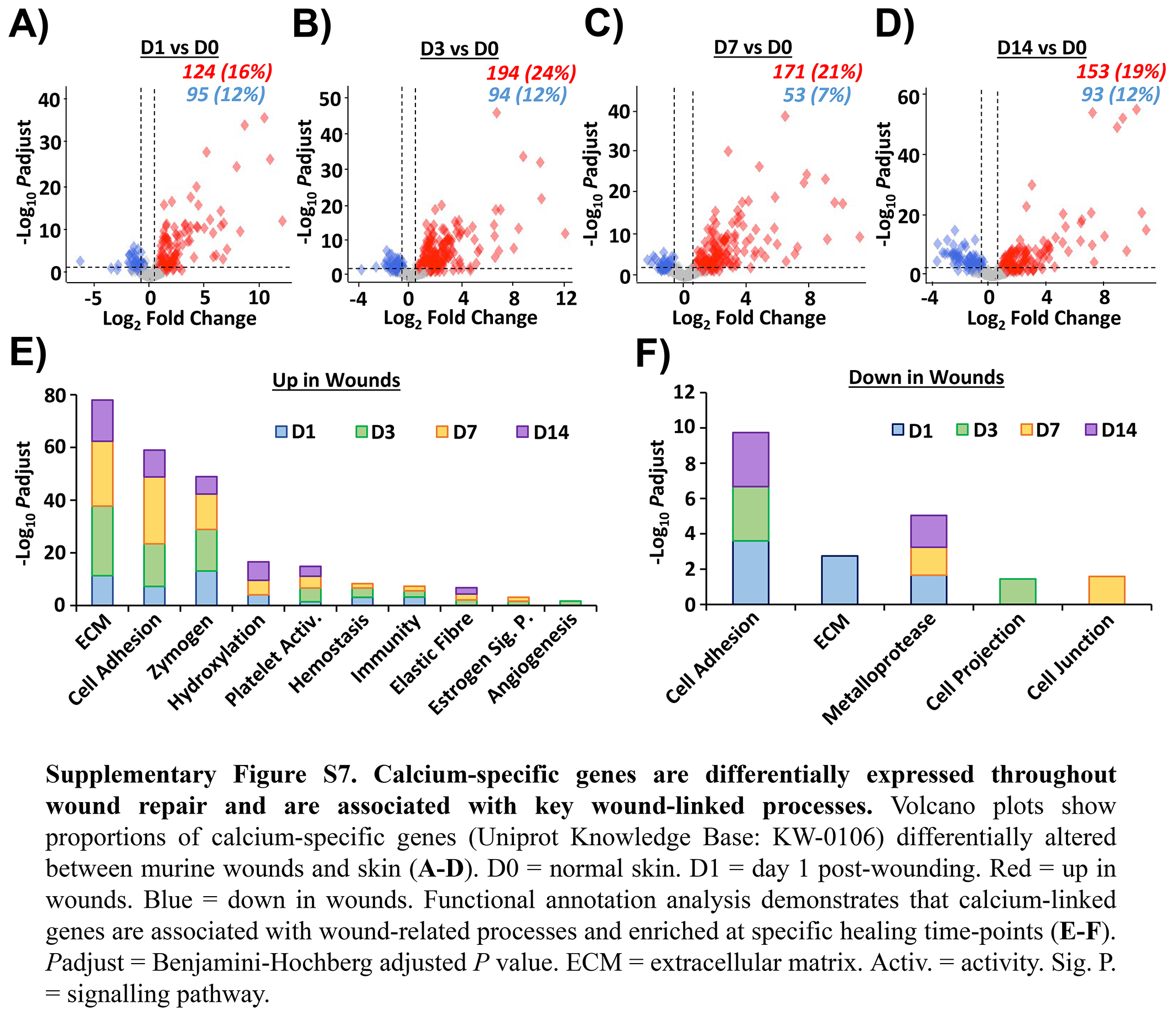

Supplement: Supplementary file 6 [file Image7.TIF]

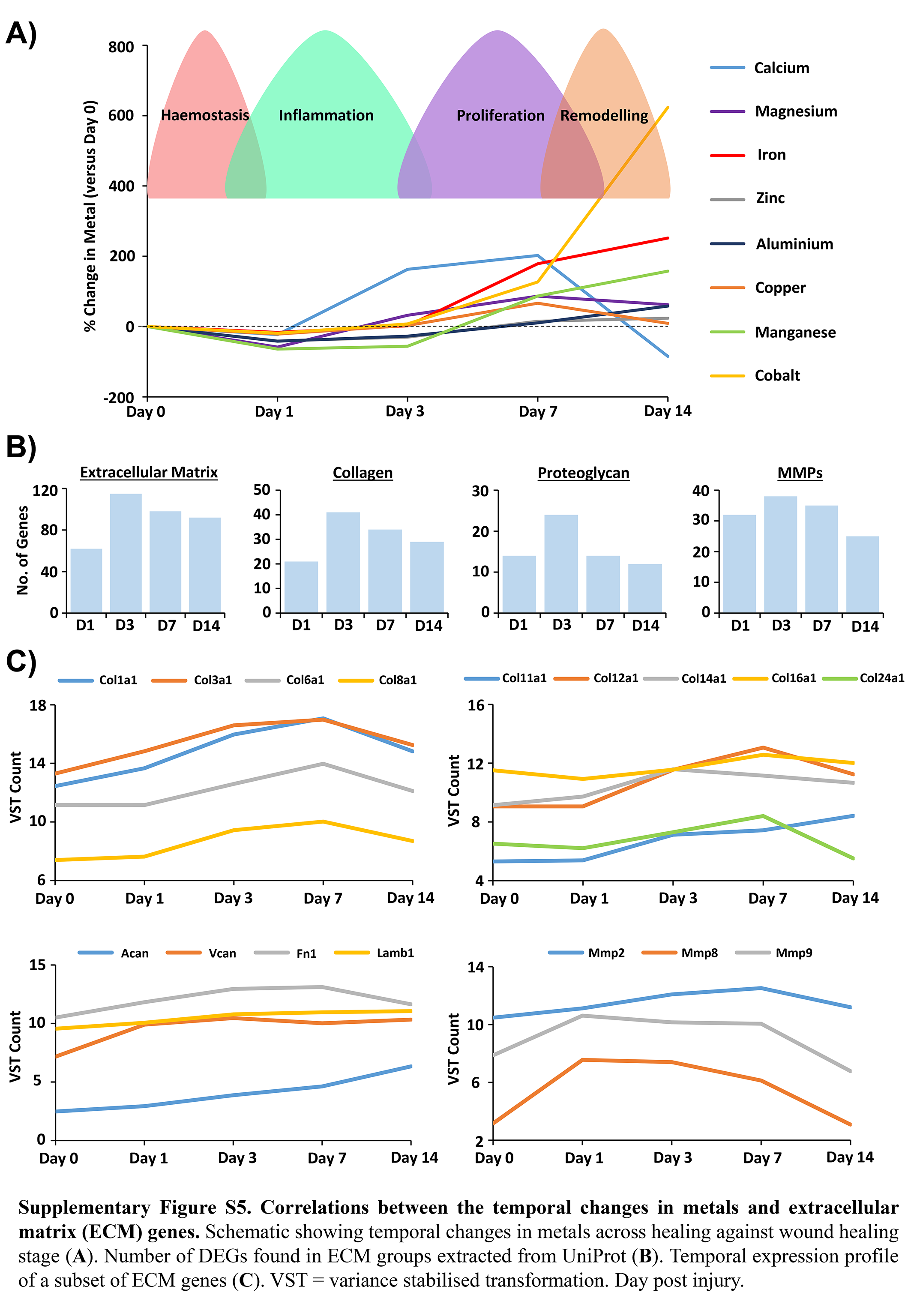

Supplement: Supplementary file 7 [file Image5.TIF]
